# RNA-seq analysis of the kidneys of broiler chickens fed diets containing different concentrations of calcium

**DOI:** 10.1038/s41598-017-11379-7

**Published:** 2017-09-18

**Authors:** Woncheoul Park, Deivendran Rengaraj, Dong-Yong Kil, Heebal Kim, Hak-Kyo Lee, Ki-Duk Song

**Affiliations:** 1 Department of Agricultural Biotechnology and Research Institute for Agriculture and Life Sciences, Seoul National University, Seoul, Republic of Korea; 2 Department of Animal Science and Technology, Chung-Ang University, Anseong, Republic of Korea; 3 Department of Animal Biotechnology and the Animal Molecular Genetics and Breeding Center, Chonbuk National University, Jeonju, Republic of Korea; 4 C&K genomics, Main Bldg. #514, SNU Research Park, Seoul, 151-919 Republic of Korea

## Abstract

Calcium (Ca) is required for normal growth and is involved in cellular physiology, signal transduction, and bone mineralization. In humans, inadequate Ca intake causes hypocalcaemia, and excessive Ca intake causes hypercalcemia. In chicken, Ca is also required for body weight gain and eggshell formation. However, transcriptomic responses to low/high Ca intake, and mechanisms affecting body weight have not been explored. In this study, we performed comparative RNA sequencing (RNA-seq) using the kidney of broiler chickens fed diets containing 0.8, 1.0, and 1.2% Ca. Annotation of RNA-seq data revealed a significant number of differentially expressed genes (DEGs) in the kidney via pairwise comparison using Cufflinks and edgeR. Using edgeR, we identified 12 DEGs; seven overlapped with those found by cufflinks. Seven DEGs were validated by real-time quantitative-PCR (qRT-PCR) in Ca-supplemented kidneys, and the results correlated with the RNA-seq data. DEGs identified by cufflinks/edgeR were subjected to pathway enrichment, protein/protein interaction, and co-occurrence analyses to determine their involvement in disease. The National Research Council (NRC) recommended Ca intake for 21-day post-hatch broilers is about 1.0%. Our findings suggest that higher-than-recommended Ca intake (1.2%) could reduce body weight gain in broilers, and that affected DEGs are related to stress-induced diseases, such as hypertension.

## Introduction

Calcium (Ca) is an essential mineral required for normal cellular physiology and signal transduction in organisms. It is mainly used for the mineralization of the endoskeleton in higher vertebrates and the exoskeleton in invertebrates. In the human body, 98% of Ca is stored in the skeleton, and only 2% is released into the extracellular fluids as calcium ions (Ca^2+^). Ca^2+^ are transported through the bloodstream either as free ions or bound with carrier proteins to the functional site. Ca is required for the formation of bone and teeth in vertebrates, shell in invertebrates, and eggshell in hard egg-laying species. Ca is involved in a wide spectrum of functions, and acts as a secondary messenger for neuromuscular signalling, contraction of the heart and muscles, hormone secretion, and as a cofactor for blood coagulation. Studies have reported that Ca has a direct effect on membrane-spanning Ca receptors, which are coupled through G proteins to intracellular signalling, and this receptor has been detected in several tissues including the parathyroid gland, kidney, brain, bone marrow, and breast^1–5^. Hypocalcaemia and hypercalcaemia are the clinical terms linked to abnormal Ca concentration in the blood. Hypocalcaemia occurs when Ca loss exceeds normal levels, in two stages: mild hypocalcaemia and severe hypocalcaemia. Hypercalcaemia occurs when Ca gain exceeds normal levels, and occurs in three stages; mild hypercalcaemia, moderate hypercalcaemia, and severe hypercalcaemia^6,7^. The symptoms of severe hypocalcaemia are associated with the loss/weakness of muscle and nerve function, milk fever in cows, and tetany in lactating cows, pigs, and dogs. In addition, lower intake of Ca is related to both hypertension and preeclampsia^8,9^, and hypercalcaemia is related to hypertension^10^. Two hormones—parathormone and calcitonin—tightly regulate Ca homeostasis. In addition, acute hypocalcaemia can lead to hypotension in experimental animals through increased vascular resistance, although the specific mechanisms involved have not been elucidated^11^. Parathormone secreted from parathyroid gland increases the blood Ca level through resorption of Ca from bone, absorption of Ca in the intestine, and reabsorption of Ca in the kidney. In contrary, calcitonin secreted from the thyroid gland reduces the blood Ca level by inhibiting the Ca resorption from bone, and inhibiting the absorption/reabsorption of Ca in the intestine/kidney^6^.

In the case of chicken, the National Research Council (NRC) recommendations state that the Ca requirement varies by age and breed. The Ca requirement for 0–3-, 3–6-, and 6–8-week-old broiler chickens is 1.0, 0.9, and 0.8%, respectively^12^. Studies have reported that the Ca requirement is 1.5% for grower chicks, but that 0.9% leads to reduced phytate digestion^13^. In addition, more than 2% of Ca intake leads to a decrease in feed intake and weight gain, and an increase in the mortality rate^14^. Ca in broiler diet is essential for strengthening the bones of the animals and for increasing productivity. Therefore, accurate estimation of Ca requirements is important to maximize broiler productivity. The current NRC recommendations of Ca for optimal growth and bone formation in broiler chickens during the 21-day post-hatch period is 1.0%^12^. However, a few studies have suggested that decreasing the Ca level in diets may improve the growth performance of broiler chickens^15,16^. This beneficial effect is associated with increased inherent phosphorus (P) utilization through the reduction of Ca phosphate formation in the intestinal tract^17^, and decreased pH of the intestinal tract, which favours digestive enzyme activity through a reduction of buffering capacity^18^. In addition, higher Ca intake may induce kidney malfunction, because poultry have a limited capacity to handle high Ca loads in the blood^19^. Furthermore, 2% Ca in the diet of 7-day-old broiler leads to hypophosphatemia and decreases the growth rate^20^.

The kidney is one of the most important organs involved in Ca homeostasis, and several genes expressed in the kidney might support this function. However, there is no information regarding the genome-wide analysis of the chicken kidney in response to Ca supplementation. In this study, we performed RNA sequencing (RNA-seq) using the kidney of broiler chickens fed diets containing three different concentrations of Ca (0.8, 1.0, and 1.2%). Quality screening and annotation of the RNA-seq reads revealed several differentially expressed genes (DEGs) in the kidney samples between 0.8 and 1.0% Ca intake, 0.8 and 1.2% Ca intake, and 1.0 and 1.2% Ca intake, and the expression of seven candidate genes was analyzed by quantitative real-time PCR (qRT-PCR). Furthermore, pathway analysis, interaction analysis, and co-occurrence analysis, identified DEGs related to reduced weight gain, and identified that oxidative stress, such as hypertension, is associated with the reduced weight gain.

## Results

### Growth performance of chickens after Ca intake

At the end of the experiment (21-days post-hatch), chickens fed diets containing 0.8% Ca showed the greatest performance for initial body weight (BW), body weight gain (BWG), feed intake (FI), and feed efficiency (FE) (P < 0.01) followed by the chickens fed diets containing 1.0% Ca (P value < 0.01). Among the treatments, chickens fed diets containing 1.2% Ca had the worst performance for BW, BWG, FI, and FE (P value < 0.01) (Table 1).

**Table 1:** Effects of dietary Ca concentration on the growth performance of broiler chickens during the 21-day post-hatch period.

Items	Dietary Ca concentration			SEM	P-value
	0.8% Ca	1.0% Ca	1.2% Ca		
Initial body weight, g	39	39	39		
21-day body weight, g	923^c^	855^b^	737^a^	18.4	<0.01
Body weight gain, g/21day	884^c^	815^b^	697^a^	18.4	<0.01
Feed intake, g/21day	1,201^c^	1,125^b^	1,036^a^	25.1	<0.01
^1^Feed efficiency, g/kg	736^b^	725^b^	673^a^	7.2	<0.01

^a,b,c^Means with different superscript letters differ at P < 0.05.

^1^Feed efficiency was calculated by dividing the body weight gain (g) by feed intake (kg).

### Quality of RNA-seq reads of the kidney of chickens after Ca intake

**Figure 1:**
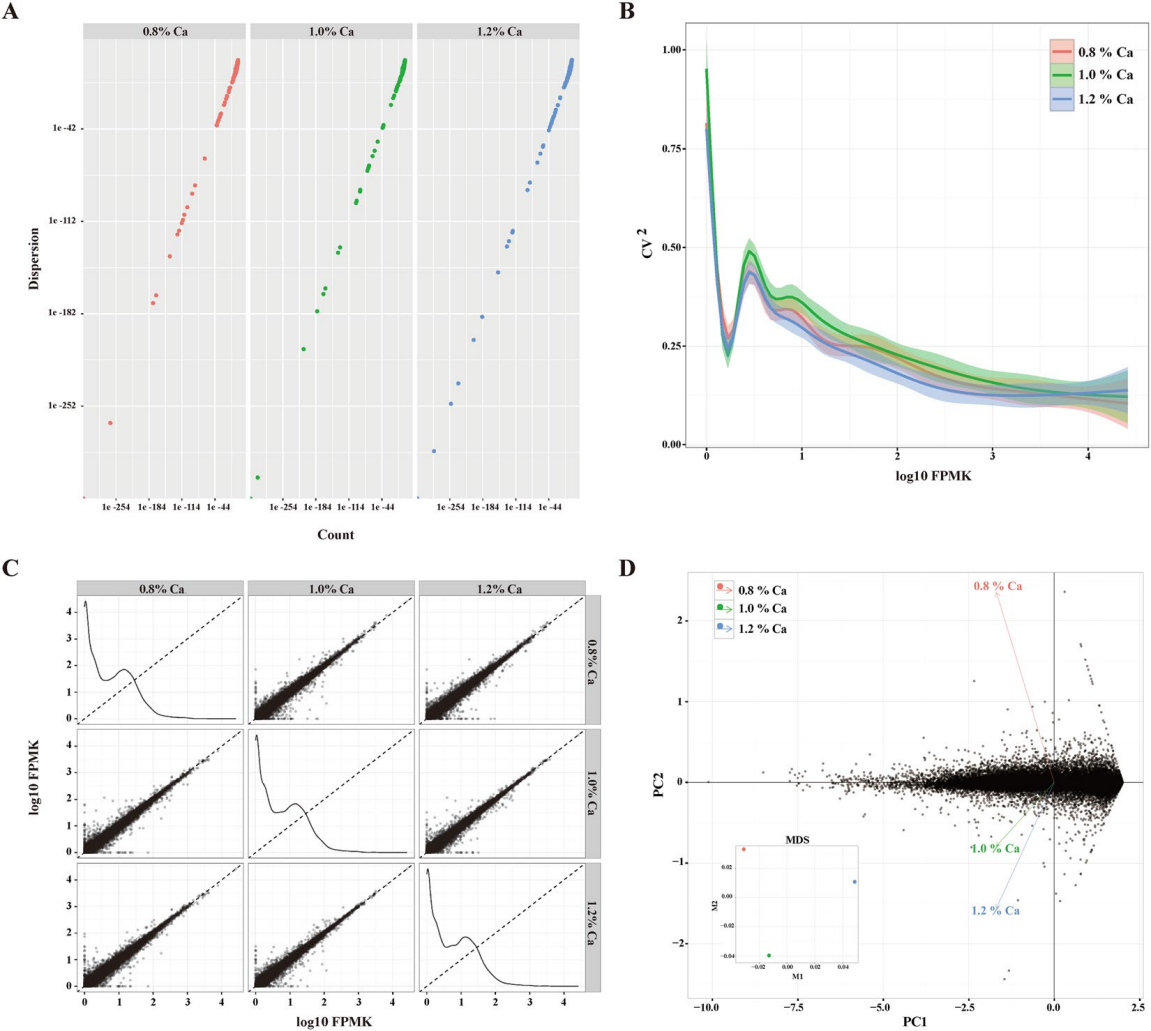
Comparative analysis of RNA-seq data from the kidney of broiler chickens fed diets containing 0.8, 1.0, and 1.2% Ca (three samples each). (**A**) Count versus dispersion plot by condition for all genes. (**B**) The squared coefficient of variation allows the visualization of cross-replicate variability between conditions, and can be a useful metric for determining data quality at the gene level. (**C**) Scatterplots to identify global changes and trends in gene expression between pairs of conditions. (**D**) PCA and MDS plots for gene-level features.

We acquired RNA-seq reads from the kidney of chickens fed diets containing 0.8, 1.0, and 1.2% Ca, which were then deposited in the NCBI Gene Expression Omnibus (Acc. No. GSE89544). The quality report for RNA-seq revealed that more than 94% of reads had an average sequencing quality score exceeding Q30. The average number of sequence reads was 10.6, 11.1, and 11.7 million in the 0.8, 1.0, and 1.2% Ca intake groups, respectively. On average, >97% sequence reads passed the trimming process using the Trimmomatic tool. In addition, most alignment rates for the three different Ca concentrations exceeded 95%, which were mapped successfully to the chicken reference genome (*Galgal4*) using Hisat2. The numbers of total sequence reads, read order, index, yield, and mapping rates for each sample are shown in Table 2. Furthermore, we used several plotting methods, such as dispersion, fpkmSCV, pairwise scatter, multi-dimensional scaling (MDS), and principal component analysis (PCA) to evaluate, cluster, and explore the quality of RNA-seq data, and the relationships between our kidney samples from animals with different Ca intake (Fig. 1).

**Table 2:** RNA-seq reads and mapping rate of kidney samples from ten chickens fed diets containing different Ca concentrations.

Lane	Sample ID	Read Order	Index	Yield (bases)	# Reads	% of > = Q30 Bases (PF)	Passed-Trimmomatic	Overall alignment rate
1	H_1_Kidney	1	CGATGT	1,183,345,896	11,716,296	94.15	11386200 (97.18%)	95.62%
1	H_1_Kidney	2	CGATGT	1,183,345,896	11,716,296	94.29		
1	M_1_Kidney	1	CCGTCC	1,002,354,199	9,924,299	93.77	9646488 (97.20%)	95.38%
1	M_1_Kidney	2	CCGTCC	1,002,354,199	9,924,299	94.26		
1	H_3_Kidney	1	ATGTCA	1,252,089,526	12,396,926	94.04	12026744 (97.01%)	95.29%
1	H_3_Kidney	2	ATGTCA	1,252,089,526	12,396,926	93.54		
1	M_2_Kidney	1	GTGAAA	1,131,942,148	11,207,348	94.10	10897037 (97.23%)	95.38%
1	M_2_Kidney	2	GTGAAA	1,131,942,148	11,207,348	94.41		
1	H_2_Kidney	1	CTTGTA	1,122,854,471	11,117,371	94.12	10798873 (97.14%)	95.49%
1	H_2_Kidney	2	CTTGTA	1,122,854,471	11,117,371	94.71		
2	L_1_Kidney	1	TAGCTT	1,013,764,270	10,037,270	94.19	9745171 (97.09%)	94.77%
2	L_1_Kidney	2	TAGCTT	1,013,764,270	10,037,270	94.57		
2	M_3_Kidney	1	TTAGGC	1,134,667,835	11,234,335	94.16	10917675 (97.18%)	92.96%
2	M_3_Kidney	2	TTAGGC	1,134,667,835	11,234,335	94.48		
2	L_4_Kidney	1	ATTCCT	1,130,748,429	11,195,529	94.25	10873936 (97.13%)	95.16%
2	L_4_Kidney	2	ATTCCT	1,130,748,429	11,195,529	94.87		
2	M_4_Kidney	1	GATCAG	1,201,298,343	11,894,043	94.29	11578895 (97.35%)	95.45%
2	M_4_Kidney	2	GATCAG	1,201,298,343	11,894,043	94.59		
2	L_3_Kidney	1	CGTACG	1,057,737,650	10,472,650	94.21	10173845 (97.15%)	95.12%
2	L_3_Kidney	2	CGTACG	1,057,737,650	10,472,650	94.03

### DEGs in the kidney of chickens after Ca intake

**Figure 2:**
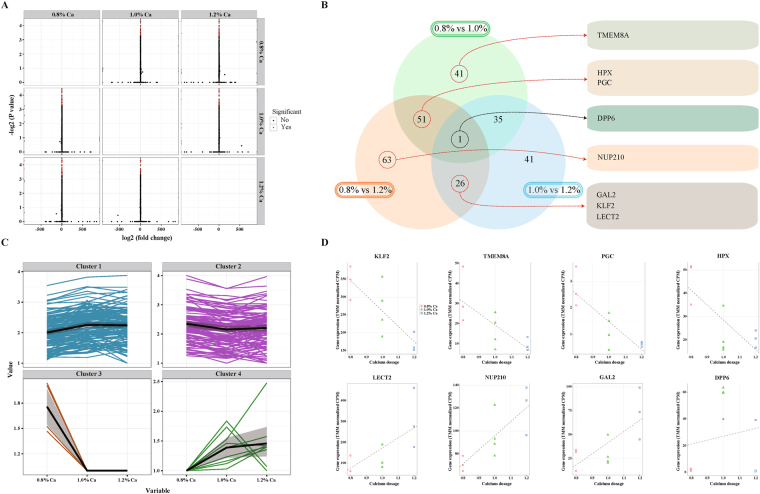
Identification of DEGs between kidney samples from broiler chickens fed diets containing 0.8, 1.0, and 1.2% Ca using both cufflinks and edgeR. (**A**) Volcano plots explore the relationship between fold-change and significance. (**B**) Venn diagram, comparing DEGs between pairs of conditions (0.8 versus 1.0, 0.8 versus 1.2, and 1.0 versus 1.2% Ca). The number of DEGs is indicated in the diagram. (**C**) Partitioning around medoids clustering with Jensen-Shannon distance for a CuffGeneSet. (**D**) Scatterplots of eight common DEGs that were identified by both cufflinks and edgeR.

After mapping the RNA-seq data to the chicken genome, we identified DEGs between the kidney samples of chicken fed diets containing 0.8, 1.0, and 1.2% Ca using two programs, cuffdiff and edgeR. First, we performed the DEG analysis between the treatment samples as follows: 0.8 and 1.0, 0.8 and 1.2, and 1.0 and 1.2% Ca using cuffdiff within cufflinks (FDR < 0.05). Thus, the numbers of DEGs between the treatment samples 0.8 and 1.0% Ca were 128 (72 upregulated, 47 downregulated, and nine infinite value). The numbers of DEGs between treatment samples 0.8 and 1.2% Ca were 141 (82 upregulated, 45 downregulated, and 14 infinite value). The numbers of DEGs between treatment samples 1.0 and 1.2% Ca were 103 (58 upregulated, 39 downregulated, and six infinite value) (Table S2). In addition, the numbers of common DEGs between 0.8 and 1.0, 0.8 and 1.2, and 1.0 and 1.2% Ca were 25, 18, and eight, respectively. Moreover, dipeptidyl peptidase-like 6 (*DPP6*) was commonly found under all conditions of the pairwise comparisons. The expression of significant DEGs in three pairwise comparisons of treatments follows four patterns (Fig. 2A–C). Second, we identified the DEGs from the association test as the likelihood ratio between three different Ca intakes and gene expression using GLM within edgeR (FDR < 0.1). Thus, 12 DEGs were identified (five upregulated and seven downregulated) (Table 3). Of these 12 DEGs, seven genes, including transmembrane protein 8 A (*TMEM8A*), progastricsin (*PGC*), hemopexin (*HPX*), nucleoporin 210 kDa (*NUP210*), Kruppel-Like factor 2 (*KLF2*), leukocyte cell derived chemotaxin 2 (*LECT2*), and galanin receptor 2 (*GAL2*) overlapped using cuffdiff (Fig. 2B and D, Table 3). Scatterplots of five DEGs identified by GLM within edgeR are shown in Figure S1.

**Table 3:** DEGs identified in the kidney from chickens fed different Ca concentrations, using the edgeR tool.

Ensembl ID	Human HGNC symbol	Chicken HGNC symbol	logFC	logCPM	LR	PValue	FDR	Association	Cufflinks overlap
ENSGALG00000003939	*KLF2*		−2.49671	8.031187	21.98473	2.75E-06	0.011116	Down regulated	8vs12, 10vs12
ENSGALG00000026188		*TMEM8A*	−4.43019	4.29225	20.94924	4.72E-06	0.014306	Down regulated	8vs10
ENSGALG00000001370	*ADAMTS8*	*ADAMTS8*	−2.1559	4.902834	19.12786	1.22E-05	0.029667	Down regulated	no
ENSGALG00000008291	*AP3S2*	*AP3S2*	−1.86177	5.509223	17.68768	2.6E-05	0.04551	Down regulated	no
ENSGALG00000028489	*PGC*		−3.33437	3.811141	17.6714	2.63E-05	0.04551	Down regulated	8vs10, 8vs12
ENSGALG00000022586	*HPX*	*HPX*	−2.94424	4.852415	16.20213	5.69E-05	0.069286	Down regulated	8vs10, 8vs12
ENSGALG00000028428			−5.30746	0.798146	15.37297	8.82E-05	0.089227	Down regulated	no
ENSGALG00000015767	*FABP4*	*FABP4*	9.578497	2.438523	23.73136	1.11E-06	0.00672	Up regulated	no
ENSGALG00000028627			9.545022	1.561843	24.04506	9.41E-07	0.00672	Up regulated	no
ENSGALG00000006323	*LECT2*	*LECT2*	3.637631	7.434093	16.70044	4.38E-05	0.06639	Up regulated	8vs12, 10vs12
ENSGALG00000005078	*NUP210*	*NUP210*	1.886229	6.587991	16.01596	6.28E-05	0.069286	Up regulated	8vs12
ENSGALG00000016669		*GAL2*	3.725195	5.388244	16.09272	6.03E-05	0.069286	Up regulated	8vs12, 10vs12

To further validate the RNA-seq data, we selected seven DEGs that primarily detected in the edgeR and overlapping in the cuffdiff program, and examined their expression in the kidney samples from the 0.8, 1.0, and 1.2% Ca intake groups by qRT-PCR. Of the selected DEGs, all seven [adaptor related protein complex 3 sigma 2 subunit (*AP3S2*), fatty acid-binding protein 4 (*FABP4*) ADAM metallopeptidase with thrombospondin type 1 motif 8 (*ADAMTS8*), *KLF2, LECT2, HPX*, and *NUP210*] were identified with the edgeR tool, however only four (*KLF2, LECT2, HPX*, and *NUP210*) were found overlapped in the cuffdiff tool. This may explain the higher accuracy and sensitivity of edgeR tool than that of cuffdiff tool. The expression patterns of selected DEGs in the RNA-seq and qRT-PCR were highly correlative (Figure S2).

### Pathway enrichment, protein/protein interaction network, and co-occurrence of DEGs

**Figure 3:**
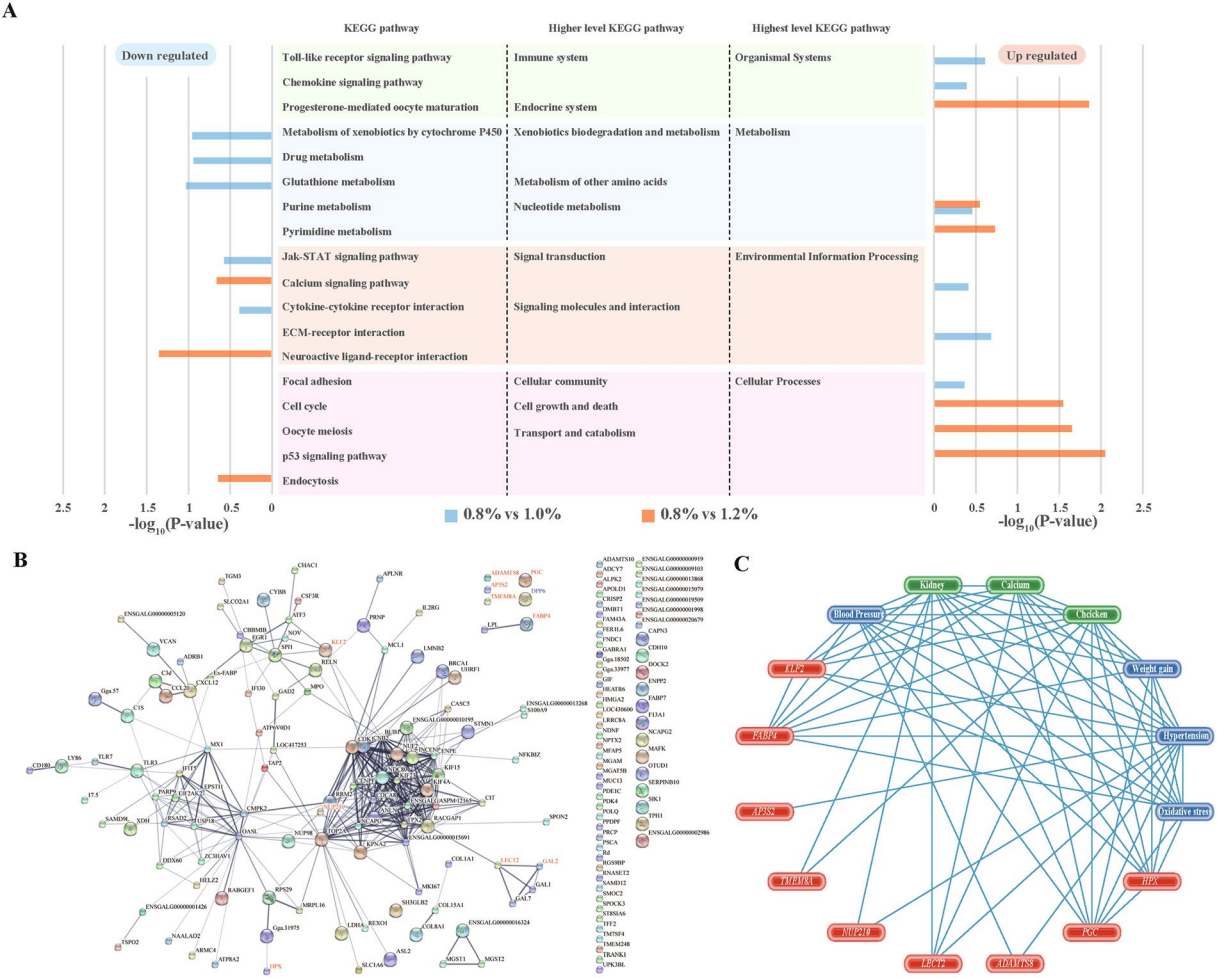
Enriched KEGG pathways, protein/protein interaction network, and co-occurrence analysis of DEGs identified between kidney samples from broiler chickens fed diets containing 0.8, 1.0, and 1.2% Ca using both cufflinks and edgeR. (**A**) KEGG pathway enrichment of DEGs identified using the cufflinks tool between pairs of conditions (0.8 versus 1.0 and 0.8 versus 1.2% Ca). (**B**) Protein/protein interactions of DEGs that were identified using the edgeR/cufflinks as generated by the STRING database. (**C**) Annotation of the co-occurrence of DEGs identified using the edgeR/cufflinks with chicken, kidney, calcium, blood pressure, hypertension, oxidative stress, and weight gain using the COREMINE database.

We used Database for Annotation, Visualization and Integrated Discovery (DAVID), Search Tool for the Retrieval of Interacting Genes/Proteins (STRING), COREMINE database, and Integrated Pathway Analysis Database for Systematic Enrichment Analysis (IPAD) database for pathway enrichment, protein/protein interaction networks, and analysis of DEGs co-occurrence in the kidney of chickens by three pairwise comparisons of Ca intake using cuffdiff. Based on the Kyoto Encyclopedia of Genes and Genomes (KEGG) pathway within the DAVID tool, downregulated DEGs between animals receiving 0.8 and 1.0% Ca were involved in the metabolism of xenobiotics by cytochrome P450, drug metabolism, glutathione metabolism, Jak-STAT signalling pathway and cytokine-cytokine receptor interaction. Upregulated DEGs between animals receiving 0.8 and 1.0% Ca were involved in the toll-like receptor signalling pathway, chemokine signalling pathway, purine metabolism, focal adhesion, ECM-receptor interaction and Ca signalling pathway. In the case of DEGs between animals receiving 0.8 and 1.2% Ca, downregulated DEGs were involved in the Ca signalling pathway, neuroactive ligand-receptor interaction, and endocytosis, and upregulated DEGs were involved in progesterone-mediated oocyte maturation, purine metabolism, pyrimidine metabolism, cell cycle, oocyte meiosis, and the p53 signalling pathway (Fig. 3A). DEGs between animals receiving 1.0 and 1.2% Ca were not found in the significant KEGG pathway. Additionally, when we checked the DEGs identified using the edgeR tool in the KEGG pathway database, six DEGs, namely *KLF2*, *ADAMTS8*, *AP3S2*, *NUP210*, *GAL2* and *FABP4*, are involved in the FoxO signalling pathway, degradation of the extracellular matrix, lysosome, RNA transport, neuroactive ligand-receptor interaction and PPAR signalling pathway, respectively (Table S3).

Next, we focused on the 12 DEGs identified using edgeR/overlapping with cuffdiff for further analysis with STRING, COREMINE, and IPAD. To explore the interaction between those five upregulated and seven downregulated DEGs identified using the edgeR/cuffdiff, we subjected them to STRING analysis for gene/protein interaction network analysis. As a result, we found there were relationships between four of five upregulated, and six of seven downregulated DEGs. In addition, four upregulated DEGs were more correlated with each other than the six downregulated DEGs (Fig. 3B and Figure S3). Figure 3B shows all the DEGs identified by edgeR and cuffdiff. Figure S3 shows only DEGs that identified from edgeR.

Based on the co-occurrence of genes and keywords (text mining) in the COREMINE database, eight DEGs (*HPX, PGC, ADMTS8, LECT2, NUP210, AP3S2, KLF2* and *FABP4*) were associated with hypertension, five DEGs (*HPX, PGC, ADMTS8, KLF2* and *FABP4*) were associated with blood pressure, four DEGs (*HPX, PGC, KLF2* and *FABP4*) were associated with oxidative stress, and three DEGs (*HPX, PGC* and *FABP4*) were associated with weight gain. *TMEM8A* gene was associated with only chicken and kidney. Moreover, chicken, kidney, Ca, hypertension, blood pressure, oxidative stress, and weight gain were most frequently associated with each other on the basis of co-occurrence (Fig. 3C). Disease information for 12 DEGs was mined in the IPAD database. As a result, only eight DEGs were found to be frequently associated with a list of 54 common diseases, including hypertension, drug toxicity, and kidney diseases (Table S4).

## Discussion

Ca is an essential mineral in general feed additive of domestic animals. The effects of Ca intake and maximum tolerable Ca intake in domestic animals have been well reported^6,14,21^. These studies define the maximum tolerable levels of minerals such as Ca, as recommended by NRC, because the NRC recommendation provides a benchmark that is useful for animal studies on minerals. The nutrient requirements of poultry were first reported in 1944 by NRC, and several updates were made by NRC up to 1994^22^. In addition, few studies are available including RNA-seq of the poultry tissue samples collected following supplementation with Ca. In the present study, we performed gene expression profiling using RNA-seq of kidney samples of broiler chickens fed diets containing three different concentrations of Ca. The aim of this study was to examine the effects of Ca, at levels recommended by NRC, on the expression of genes in the chicken kidney. DEGs were identified by pairwise comparison of treatments using the cufflinks and edgeR tools, in order to obtain reliable DEGs found by both pairwise comparison and ordinal analysis^23^. In the present study, 372 DEGs were identified among the pairwise comparisons between 0.8 and 1.0% Ca, 0.8 and 1.2% Ca, and 1.0 and 1.2% Ca using cufflinks. We identified 12 DEGs using edgeR. Of these 12 DEGs, seven DEGs, including *TMEM8A, PGC, HPX, NUP210, KLF2, LECT2*, and *GAL2* were identified by both tools. Pathway analysis of DEGs identified using cufflinks showed an opposite tendency in Ca signalling in the comparison between the 0.8 and 1.0% and 0.8 and 1.2% Ca treatments. This result suggests that 1.2% Ca intake could have an adverse effect on broiler kidneys. STRING analysis of the DEGs identified using edgeR found that DEGs with expression that increased in a linear way (*FABP4, GAL2, LECT2* and *NUP210*) were more correlated than linearly decreased DEGs (*KLF2, HPX, ADAMTS-8, TMEM8A, AP3S2* and *PGC*). Text mining edgeR-identified DEGs using the COREMINE and IPAD databases revealed that many DEGs were associated with chicken, kidney, Ca, and several common diseases. However, we mainly focused on weight gain, hypertension, oxidative stress, and blood pressure. Weight gain is an important factor in the broiler industry, and stress-induced hypertension has an impact on weight gain.

The results of our study revealed adverse outcomes, such as decreases in the initial BW, BWG, FI, and FE, in chickens fed high Ca. This finding was consistent with that of Sebastian *et al*.^16^, who reported that a high (1.25%) concentration of Ca in diets decreases the BW and FI of broilers compared with low (0.6%) or NRC-recommended (1.0%) Ca intake. Rama Rao *et al*.^15^ also reported that decreasing Ca intake from 0.9 to 0.6% increased the BW and FI of broilers. Therefore, high Ca intake can adversely affect the growth performance of chickens, and this cannot be well explained by nutritive experiments alone, because the molecular mechanism is not known. For this reason, we analyzed gene expression using RNA-seq in the broiler kidney, and then aimed to explore the molecular mechanisms of DEGs identified using edgeR, based on the available literature for humans, rodents, and poultry. We found that the expression of *FABP4, GAL2, LECT2, NUP210*, and *DPP6* increased linearly as the concentration of Ca increased. FABP4, which encodes fatty acid binding protein, was expressed in adipocytes. Fatty acid binding proteins are cytoplasmic proteins that bind unsaturated long-chain fatty acids and can reversibly bind hydrophobic ligands. In humans, increased plasma concentrations of *FABP4* have been associated with atherosclerosis, cardiac diastolic dysfunction, hypertension, insulin resistance, and obesity^24–27^. Enhanced expression of *FABP4* correlates with increased expression of CD36, CD68, CD52, CD163, and T-cell markers^28^. This gene is actively released from human adipocytes *in vitro* via a non-classical, Ca-dependent mechanism as well as via coronary artery Ca^29,30^. The expression of this gene is higher in hypertensive patients than in normotensive individuals^31^. Knockdown of this gene in disrupted nutrient metabolism in diet-induced obese mice significantly increased BW and fat mass^32^, and increased expression leads to decreased weight gain in epididymal adipose tissue of rats fed a fructose-rich diet^33^. The *LECT2* gene has been associated with adrenal amyloid and primary aldosteronism, which have no or few symptoms. such as muscular weakness, high blood pressure, and headaches^34,35^. This gene was isolated as a neutrophil-chemotactic factor produced by T cells^36^. Increased *LECT2* gene expression is specific to tumours induced by β-catenin, and activation of the *LECT2* gene can lead to the development of tumours with a better prognosis^37,38^. Recently, the product of this gene has been referred to as a recently discovered hepatokine, which mediates obesity-related metabolic disturbances and insulin resistance^39–41^. Additionally, hepatokine *LECT2* amyloidosis has been related to portal hypertension^42^. Those studies note that increased *LECT2* expression is commonly observed in insulin resistance and obesity in human and mouse. However, the role of *LECT2* in the development of obesity and insulin resistance induced by over-nutrition has not yet been established. The *NUP210* gene has been associated with diseases, such as autoimmune disease of the urogenital tract and primary biliary cirrhosis (PBC), and PBC is related to pulmonary hypertension and polymyositis^43,44^. In addition, anti-NUP210 antibodies have been used to diagnose PBC with jaundice and liver failure^45^. The *NUP210* gene was initially identified as an early-response gene in metanephric kidney development in mouse^46^. The *GAL2* gene encodes a galactose permease, and is required for the utilization of galactose and the transport of glucose^47–49^. In addition, *GAL2* was found to be upregulated in whole blood cells of wild passerine following immune stimulation with lipopolysaccharides^50^. Expression of *DPP6* has been associated with ischemic heart disease^51^. Based on those findings, we suggest that the linear increase in *FABP4* and *LECT2* expression with increasing Ca concentrations in broiler kidney could lead to more T-cells being activated, stimulate Ca-dependent mechanisms, exhibit a protective effect in tumourigenesis, and directly induce hypertension. Moreover, *FABP4* gene expression was negatively correlated with BW and fat mass, whereas *LECT2* gene expression was positively correlated with obesity and insulin resistance. Thus, we speculated that high Ca intake decreases BWG and FI in broilers, even if they show obesity and high insulin resistance. However, we are unable to corroborate a direct effect for *NUP210, GAL2* and *DPP6* on blood pressure and hypertension, but we suggest that these genes could be indirectly related to blood pressure and hypertension.

We found that the expression *KLF2, HPX, ADAMTS-8, TMEM8A, AP3S2* and *PGC* decreased linearly as the concentration of Ca increased. *KLF2* is a member of the Krüppel-like factor family, which include broadly expressed zinc finger transcription factors. This has been associated with the lung development, embryonic erythropoiesis, epithelial integrity, T-cell viability, adipogenesis, B-cell homeostasis, plasma cell homing and vascular growth/remodelling^52–54^. Overexpression of this gene in human and mouse increase the number of proangiogenic cells (PACs) *in vitro*, and improved the neovascularization abilities of aged mouse PACs in an ischemic hind limb model *in vivo*, respectively^55^. However, the number of PACs and the neovascularization abilities are disrupted by risk factors for ischemic heart disease, such as age, hypertension and smoking^56^. Expression of this gene in the developing chick heart was decreased by trichloroethylene, which may function to alter endothelial development^57^. Expression of this gene was decreased in areas of low and disturbed wall shear stress, which is related to blood flow in heart development^58^. Therefore, increased expression of the *KLF2* gene improves portal hypertension^59^. Moreover, this gene is an adipogenesis inhibitor, and increased expression of this gene induced by retinoic acid prevents diet-induced weight gain^60^. *HPX* encodes a haem-binding protein, and the structure of the chicken *HPX* gene is more complex than that of the mammalian *HPX* gene. This gene shows an acute phase response in chicken, and its expression is increased during infection^61^. *HPX* expression is decreased in idiopathic intracranial hypertension and preeclampsia, which are associated with symptoms such as hypertension, pitting oedema, epigastric pain and swelling^62^. Moreover, this gene has been associated with daily weight gain and backfat thickness in large white pigs by protein phenotypes, and has also been associated with susceptibility or resistance to diet-induced obesity^63,64^. There is evidence that the *TMEM8A* gene is also associated with preeclampsia^65^. *ADAMTS-8* encodes a member of the ADAMTS protein family and aggrecanases. *ADAMTS-8* was found to influence pulse pressure and mean arterial pressure by a genome-wide association study^66,67^. *AP3S2* gene expression is associated with carotid plaques and obesity in individuals with type 2 diabetes mellitus^68–70^. Therefore, our results suggest that the linear decrease in *KLF2, HPX, TMEM8A, ADAMTS-8* and *AP3S2* expression in the broiler kidney is directly or indirectly related to blood pressure, hypertension and weight gain. However, we are unable to state this for the *PGC* gene, because we could not find clear evidence for an effect on blood pressure with hypertension and weight gain.

## Conclusions

In this study, we demonstrated that Ca increase leads to reduced BWG and FI using pathway enrichment, protein-association networks, and co-occurrence analysis of DEGs identified using the cufflinks and edgeR tools in the kidney of chickens fed different levels of Ca. First, we identified DEGs that are directly related to weight gain. Second, we identified DEGs that are related to stress-induced disease, such as hypertension, which affects weight gain. Although a few of these DEGs have not been previously reported in relation to blood pressure, hypertension, and weight gain, we suggest that they may play a role in blood pressure, with hypertension and weight gain. However, additional studies should investigate their roles and functions. Our findings contribute to a better understanding of the potential molecular mechanisms underlying the correlation between Ca intake, BWG, FI, and stress-induced hypertension in broiler chickens. We suggest that the maximum tolerable Ca intakes should be a level between 0.8–1.0%, and increasing to over 1% is not advisable for normal growth of broilers.

## Methods

### Experimental birds and care

Ross 308 broiler chicks obtained from Yangji hatchery (Pyeongtaek, Korea) were used in this study. The care and experimental use of birds was reviewed and approved by the Institutional Animal Care and Use Committee at Chung-Ang University (IACUC No.: 14-0005). Animal management, treatment, sample collection, and further analysis of RNA-seq data and qRT-PCR were performed in either Chung-Ang University or Chonbuk National University-affiliated laboratories. All animal experiments were performed in accordance with the relevant guidelines and regulations.

### Experimental design and sample collection

In total, 1,280 one-day-old Ross 308 broiler chickens (initial BW = 39.4 ± 0.17 g) were allotted to one of three dietary treatments with six replicates, with each replicate consisting of 70 birds, in a completely randomized design. Chickens were housed in conventional floor pens (200-cm width × 230-cm length × 100-cm height for each pen) for 21 days. Three commercial-type experimental diets were formulated, and the concentrations of Ca in the diets were 0.8, 1.0, and 1.2% each. The concentrations of non-phytate phosphorus (NPP) in all diets were maintained at 0.35%, and all diets were supplemented with commercial phytase (Phyzyme XP, Danisco Animal Nutrition, Marlborough, UK) at 1,000 FTU/kg. All diets were formulated to meet or exceed the NRC (1994) requirements for broiler chickens during the first 21 days post hatch, with the exception of Ca and NPP. All birds were provided with diets fed in mash form and water ad libitum. The room temperature was maintained at 30 °C during the first week and then gradually decreased to 24 °C at the end of the experiment. A 24-h photoperiod was used throughout the experiment. The BWG and FI were recorded at the end of the experiment. Feed efficiency (g/kg) was calculated by dividing BWG with FI. At the end of the experiment (21-days post-hatch), four chickens per treatment with a BW close to the treatment mean BW were euthanized by CO2 asphyxiation and immediately dissected. The kidney samples were collected, frozen using liquid nitrogen, and kept in a freezer at −80 °C until further analysis.

### RNA sequencing and library preparation

Total RNA from the kidney samples (50–100 mg) was isolated from 10 broiler chickens (0.8% = three chickens, 1.0% = four chickens and 1.2% = three chickens) using the TRIzol (Invitrogen, USA) reagent for the sequencing and construction of a RNA-seq library. For RNA-seq, the TruSeq RNA Sample Pre Kit was used according to the manufacturer’s guidelines. Agilent Technologies Human UHR total RNA was used as a positive control sample. The library was constructed according to a standard protocol provided by Illumina, Inc. Libraries with different indexes were pooled and sequenced in one lane using an Illumina HiSeq. 2000 high-throughput sequencing instrument with 100 paired-end (PE) reads.

### Alignment of raw reads to the chicken transcriptome

After RNA-seq, the reads were trimmed to remove the adapter sequence, the specific sequence of the other Illumina, and reads less than 80 base pairs (bp) using the Trimmomatic ver 0.32 tool^71^. Subsequently, the reads were aligned with the chicken (*Gallus gallus*) reference genome obtained from the Ensembl website (ftp://ftp.ensembl.org/pub/release-85/fasta/gallus_gallus/dna/) using Hisat2 ver 2.0.4 tool^72^. Hisat2 is a fast and sensitive alignment tool for mapping next-generation sequencing reads. During analysis through Hisat2, we used the default options, and added the–dta-cufflinks option, which report alignments tailored specifically for Cufflinks. Next, we used the Featurecount tool^73^ to count the number of reads for each gene.

### Differentially expressed genes analysis

DEGs were identified using Cufflinks ver 2.2.1^74^ and R package edgeR^75^. First, we used the Cufflinks tool following the default options, and added the -g/–GTF-guide, which finds novel genes and isoforms by Reference Annotation Based Transcript (RABT) assembly. Next, cuffdiff within cufflinks uses the normalized RNA-seq fragment to estimate transcript abundance. Fragments per kilobase of exon per million fragments mapped (FPKM) of each sample were counted to estimate the expression levels of the transcripts. Cuffdiff was used to estimate the differential expression of transcripts across condition points and identify significant changes in gene expression. Since cuffdiff can detect DEGs between two samples, we compared DEGs in the kidney samples from the 0.8 and 1.0% Ca intake, 0.8 and 1.2% Ca intake, and 1.0 and 1.2% Ca intake groups. Significant DEGs were identified by a false discovery rate (FDR) of <0.05. Moreover, cummeRbund^76^ was used for the visualization and exploration of cuffdiff results. Second, we used the R package edgeR, which is based on a negative binomial model and count data. When a negative binomial model is used, the dispersion should be computed before DEGs analysis is implemented. Generalized linear models (GLM) state the probability distributions based on the relationship between mean and variance. The GLM likelihood ratio test is based on the idea of fitting negative binomial GLMs with Cox-Reid dispersion estimates. This automatically takes all known sources of variation into account. Significant DEGs were detected with a cut-off value of FDR < 0.1.

### Quantitative real-time PCR analysis

qRT-PCR analysis was performed to validate the expression of seven DEGs between the Ca treatments, and the results were screened using both cuffdiff and edgeR (four genes), or edgeR alone (three genes) (Table S1 Total RNA (1 μg) from the kidneys of chickens fed three different Ca concentrations was reverse-transcribed using SuperScript™ III cDNA synthesis kit (Thermo Fisher Scientific) according to manufacturer’s guidelines. cDNA was then amplified using TaqMan PreAmp Master Mix Kit (Applied Biosystems, Foster City, CA, USA) according to the manufacturer’s protocol. PCR was performed in an ABI PRISM 7900HT Sequence Detection System (Applied Biosystems). The PCR mix consisted of 10 μL 2 × TaqMan Fast Universal PCR Master Mix, No AmpErase UNG, 1 μL 0.2 μM TaqMan probe, 3 μL 1.5 μM forward primer, 1.4 μL 0.7 μM reverse primer, and 1.33 μL of cDNA to a final volume of 20 μL. The PCR was initiated with 10-minutes incubation at 95 °C, followed by 40 cycles of 95 °C for 15 seconds and 60 °C for 60 seconds. All samples were amplified in triplicate and the data were analysed with Sequence Detector software (Applied Biosystems). The gene expression was calculated after normalization to chicken glyceraldehyde-3-phosphate dehydrogenase gene (*GAPDH*), and using the 2^−ΔΔCt^ method^77^.

### Pathway enrichment analysis

The chicken Ensembl gene IDs were converted into official gene symbols by cross matching to human Ensembl gene IDs, and to official gene symbols using the DAVID program^78^. The official gene symbols of human homologues of chicken genes were then used for functional clustering and enrichment analyses using the DAVID program. The representation of functional groups in three pairwise comparison treatments, as 0.8 and 1.0, 0.8 and 1.2, and 1.0 and 1.2% Ca intake relative to the whole genome, was investigated using the Expression Analysis Systematic Explorer (EASE) tool^79^ within DAVID. Pathway analysis of DEGs was carried out using the KEGG tool. To identify enriched KEGG pathways, functionally clustered genes were filtered by EASE value < 0.1.

### Protein/protein interaction network and Co-occurrence analysis

We used the STRING database^80^ to construct the protein/protein interaction network. During the STRING analysis, we set parameters to include proteins with at least one connection, and a medium confidence score (≥0.4). In addition, we used text mining methods to screen the DEGs related to hypertension and blood pressure by the COREMINE^81^ and the IPAD^82^ databases, respectively. The DEGs and the exact keywords hypertension, blood pressure, chicken, kidney, and Ca were input into COREMINE for co-occurrence analysis, and disease information among DEGs was mined in the IPAD database.

### Accessions codes

The raw RNA-seq data obtained from the kidney of broiler chickens fed diets containing 0.8, 1.0, and 1.2% Ca were deposited at the NCBI Gene Expression Omnibus (https://www.ncbi.nlm.nih.gov/geo/) under the accession GSE89544.

## Electronic supplementary material

Supplementary Figure and Table
