# Supplementary material for: RNA-seq analysis of the kidneys of broiler chickens fed diets containing different concentrations of calcium
Source: Sci Rep. 2017 Sep 18;7:11740. doi: 10.1038/s41598-017-11379-7 (PMC5603577; doi:10.1038/s41598-017-11379-7)
Supplement: Supplementary file 1 — Supplementary Figure and Table [file 41598_2017_11379_MOESM1_ESM.docx]

**Supplementary Information for**

**RNA-seq analysis of the kidneys of broiler chickens fed diets containing different concentrations of calcium**

Woncheoul Park^1,4, †^, Deivendran Rengaraj^2,^ ^†^, Dong-Yong Kil^2^, Heebal Kim^1^, Hak-Kyo Lee^3,*^, Ki-Duk Song^3,*^

^1^Department of Agricultural Biotechnology and Research Institute for Agriculture and Life Sciences, Seoul National University, Seoul, Republic of Korea

^2^Department of Animal Science and Technology, Chung-Ang University, Anseong, Republic of Korea

^3^Department of Agricultural Biotechnology and the Animal Molecular Genetics and Breeding Center, Chonbuk National University, Jeonju, Republic of Korea

^4^C&K genomics, Main Bldg. #514, SNU Research Park, Seoul 151-919, Republic of Korea

^†^ These authors equally contributed and should be regarded as co-first authors.

*Corresponding author:

Hak-Kyo Lee ([breedlee@empas.com](mailto:breedlee@empas.com), TEL : +82-63-270-4748, Fax : +82-63-270-4614)

Ki-Duk Song (kiduk.song@gmail.com , TEL : +82-63-219-5523, Fax : +82-63-270-5937)

**Table of contents**

**Supplementary Fig. S1-S3**

Supplementary Fig. S1 | Scatterplots of 5 DEGs that were identified by only edgeR tool.

Supplementary Fig. S2 | qRT-PCR validation of DEGs identified using either the cufflinks and edgeR tools or edgeR alone.

Supplementary Fig. S3 | Protein/protein interaction network of DEGs (4 upregulated and 6 downregulated) identified using the edgeR/cufflinks.

**Supplementary Tables S1-S4**

Supplementary Table S1 | The primer sequences of DEGs used for qRT-PCR analysis.

Supplementary Table S2 | List of DEGs identified from pairwise comparison among three different Ca intake using the cufflinks tool.

Supplementary Table S3 | Enriched KEGG pathways of the DEGs identified using the edgeR tool.

Supplementary Table S4 | Annotation of the co-occurrence of DEGs identified using the edgeR/cufflinks tools with IPAD database.

**

Supplementary Figure S1. Scatterplots of 5 DEGs that were identified by only edgeR tool.** Of these 5 DEGs, 2 were upregulated (ENSGAL00000028627 and FABP4) and 3 were downregulated (ENSGAL00000028428, ADAMTS8, and AP3S2).


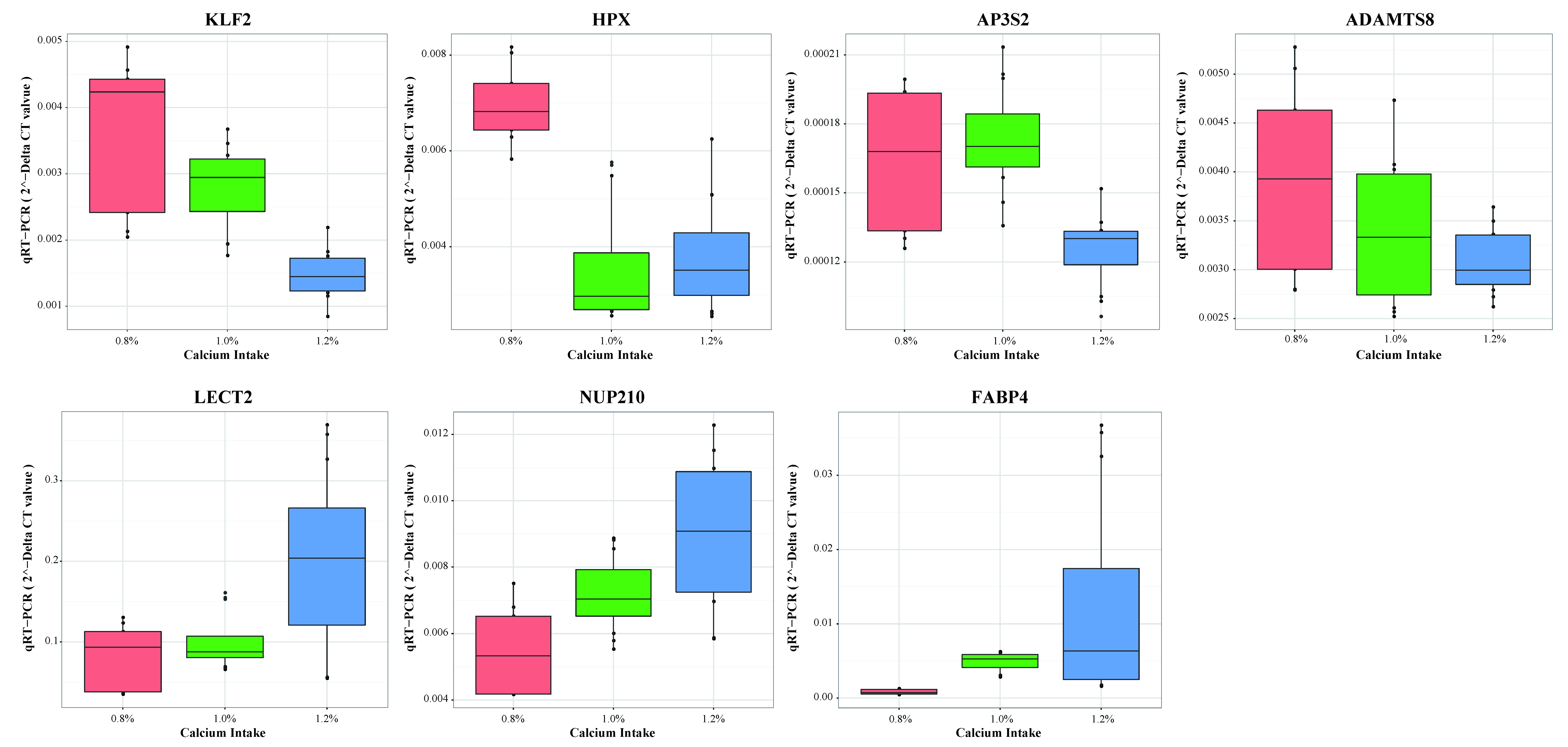
**Supplementary Figure S2. qRT-PCR validation of DEGs identified using either the cufflinks and edgeR tools or edgeR alone.** *KLF2, HPX, LECT2,* and *NUP210* were identified by both cufflinks and edgeR tools. *AP3S2, ADAMTS8,* and *FABP4* were identified by only edgeR tool.


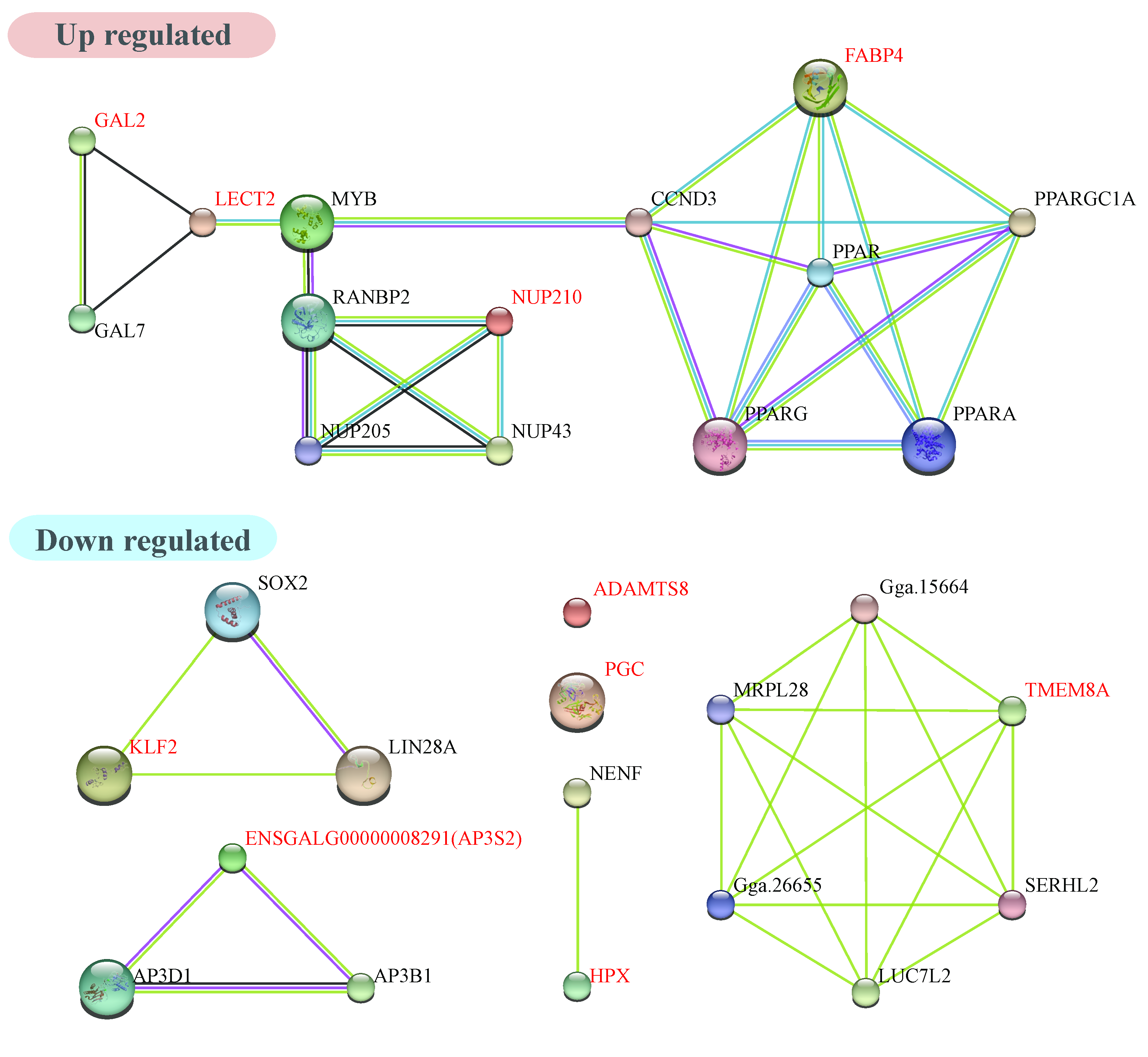
**Supplementary Figure S3. Protein/protein interaction network of DEGs (4 upregulated and 6 downregulated) identified using the edgeR/cufflinks.** This interaction network was prepared using STRING, and the parameters set were proteins with at least 10 connection and a medium confidence scores (>=0.4).

**Supplementary Table S1. The primer sequences of DEGs used for qRT-PCR analysis.**

| **Gene** | **Gene full name?** | **Ensembl Accession No.** | **Primer sequence (5` to 3`)** | |
| --- | --- | --- | --- | --- |
| *KLF2* |  | ENSGALG00000003939 | F | CCCACCTGCGGACACA |
|  |  |  | R | CAGCCCTCCCAGTTGCA |
| *HPX* |  | ENSGALG00000022586 | F | GCCGAGGGCACAGACAT |
|  |  |  | R | ACTGCAGCGGTCACCAG |
| *LECT2* |  | ENSGALG00000006323 | F | GATACGGCTGCGGCAATTAC |
|  |  |  | R | GCCCTTGTGCTTTTCTCCTTT |
| *NUP210* |  | ENSGALG00000005078 | F | GTCTCATCTCAAGGCAGCTAAAGTA |
|  |  |  | R | GGTGGCAGACACTGGTAGAA |
| *AP3S2* |  | ENSGALG00000008291 | F | CGGCTCGTCCGCTTCTAC |
|  |  |  | R | CCGCAGCACCATAGAAGGT |
| *ADAMTS8* |  | ENSGALG00000001370 | F | GCACTATGACACTGCCATCCT |
|  |  |  | R | CGTGTCGCAGCCTTGATG |
| *FABP4* |  | ENSGALG00000015767 | F | CCTGGAAGCTCCTTTCTAGTGAAAA |
|  |  |  | R | CAGCCATCTTCCTGGTAGCAAA |
| *GAPDH* |  | ENSGALG00000014442 | C | TCGTCAAGCTTGTTTCCTGGTATGA |

**Supplementary Table S2. List of DEGs identified from** **pairwise comparison among three different Ca intake using the cufflinks tool.**

| **Gene_id** | **Gene** | **Locus** | **S1** | **S2** | **value_1** | **value_2** | **log2(FC)** | **test_stat** | **p_value** | **q_value** | **Sig** |
| --- | --- | --- | --- | --- | --- | --- | --- | --- | --- | --- | --- |
| XLOC_000180 | HMGI-C | 1:34166423-34271956 | 0.8% | 1.0% | 3.8466 | 17.7798 | 2.20859 | 4.04216 | 0.0002 | 0.026681 | yes |
| XLOC_000282 | APOLD1 | 1:48046727-48051925 | 0.8% | 1.0% | 86.0232 | 53.8495 | -0.67579 | -2.12951 | 5.00E-05 | 0.00817 | yes |
| XLOC_000439 | ENSGALG00000013057 | 1:62212512-62227128 | 0.8% | 1.0% | 13.432 | 27.0429 | 1.00958 | 2.93832 | 5.00E-05 | 0.00817 | yes |
| XLOC_000650 | NFKBIZ | 1:84799573-84816130 | 0.8% | 1.0% | 19.2603 | 30.9981 | 0.686554 | 2.2303 | 5.00E-05 | 0.00817 | yes |
| XLOC_000761 | Mx | 1:108275627-108317944 | 0.8% | 1.0% | 23.8275 | 48.0971 | 1.01332 | 2.25703 | 5.00E-05 | 0.00817 | yes |
| XLOC_001175 | HPX | 1:193596424-193599483 | 0.8% | 1.0% | 27.0104 | 12.8102 | -1.07623 | -2.49347 | 5.00E-05 | 0.00817 | yes |
| XLOC_001286 | RELN | 1:12856182-13129087 | 0.8% | 1.0% | 6.68483 | 13.7875 | 1.0444 | 3.54369 | 5.00E-05 | 0.00817 | yes |
| XLOC_001661 | ZC3HAV1 | 1:71546271-71571605 | 0.8% | 1.0% | 19.24 | 29.7437 | 0.628477 | 2.01477 | 0.0002 | 0.026681 | yes |
| XLOC_001690 | MFAP5 | 1:75420698-75444541 | 0.8% | 1.0% | 38.3903 | 69.526 | 0.856808 | 2.95155 | 5.00E-05 | 0.00817 | yes |
| XLOC_001787 | COL8A1 | 1:83661752-83748495 | 0.8% | 1.0% | 23.3474 | 35.4521 | 0.602608 | 2.01885 | 0.0002 | 0.026681 | yes |
| XLOC_001928 | Nox2 | 1:112488845-112516983 | 0.8% | 1.0% | 23.5703 | 13.6242 | -0.79079 | -2.02907 | 0.00025 | 0.031299 | yes |
| XLOC_002268 | PRCP | 1:188219968-188248663 | 0.8% | 1.0% | 25.576 | 15.9995 | -0.67677 | -2.1938 | 5.00E-05 | 0.00817 | yes |
| XLOC_002611 | - | 1:153879687-153881865 | 0.8% | 1.0% | 1.92923 | 0 | #NAME? | #NAME? | 5.00E-05 | 0.00817 | yes |
| XLOC_003525 | ADCY7 | 11:6008011-6066905 | 0.8% | 1.0% | 44.8387 | 71.6893 | 0.677013 | 2.33165 | 5.00E-05 | 0.00817 | yes |
| XLOC_003918 | OAS*A | 12:3221939-3228494 | 0.8% | 1.0% | 42.5503 | 102.809 | 1.27272 | 3.56557 | 5.00E-05 | 0.00817 | yes |
| XLOC_004290 | ENSGALG00000026654 | 13:16565757-16577228 | 0.8% | 1.0% | 22.6911 | 4.48386 | -2.33931 | -3.20075 | 5.00E-05 | 0.00817 | yes |
| XLOC_004307 | EGR1 | 13:17704809-17707751 | 0.8% | 1.0% | 115.325 | 227.376 | 0.979367 | 2.97902 | 5.00E-05 | 0.00817 | yes |
| XLOC_004353 | GABRA1 | 13:6492603-6646067 | 0.8% | 1.0% | 1.81189 | 5.32276 | 1.55468 | 2.8113 | 5.00E-05 | 0.00817 | yes |
| XLOC_004365 | - | 13:7697747-7705169 | 0.8% | 1.0% | 25.5474 | 40.5616 | 0.666936 | 2.07944 | 0.00015 | 0.021491 | yes |
| XLOC_004690 | ENSGALG00000026188 | 14:12104177-12121865 | 0.8% | 1.0% | 4.64661 | 1.54558 | -1.58803 | -2.913 | 5.00E-05 | 0.00817 | yes |
| XLOC_004691 | melanoma | 14:12121978-12125813 | 0.8% | 1.0% | 7.38947 | 2.36271 | -1.64503 | -2.35191 | 0.00025 | 0.031299 | yes |
| XLOC_005330 | ENSGALG00000021139 | 15:7928723-7935907 | 0.8% | 1.0% | 340.073 | 137.292 | -1.3086 | -3.50543 | 5.00E-05 | 0.00817 | yes |
| XLOC_005565 | ENSGALG00000008518 | 17:1933341-1940628 | 0.8% | 1.0% | 34.5361 | 18.3108 | -0.91541 | -2.57335 | 5.00E-05 | 0.00817 | yes |
| XLOC_005601 | LRRC8A | 17:5305567-5310846 | 0.8% | 1.0% | 4.25121 | 0.918916 | -2.20987 | -2.87711 | 5.00E-05 | 0.00817 | yes |
| XLOC_005723 | Ex-FABP | 17:501841-504845 | 0.8% | 1.0% | 1009.52 | 357.186 | -1.49892 | -4.8844 | 5.00E-05 | 0.00817 | yes |
| XLOC_005725 | PTGDS | 17:509027-511253 | 0.8% | 1.0% | 78.0361 | 33.0126 | -1.24113 | -3.13169 | 5.00E-05 | 0.00817 | yes |
| XLOC_005790 | SH3GLB2 | 17:5432743-5457800 | 0.8% | 1.0% | 1.42784 | 4.54423 | 1.6702 | 2.47968 | 0.0001 | 0.015354 | yes |
| XLOC_006305 | RABGEF1,TMEM248,gga-mir-6585 | 19:5031339-5068523 | 0.8% | 1.0% | 84.7961 | 151.718 | 0.83932 | 3.34037 | 5.00E-05 | 0.00817 | yes |
| XLOC_006481 | CRYD2 | 19:4932192-4941557 | 0.8% | 1.0% | 19.775 | 11.2569 | -0.81286 | -1.94518 | 0.0002 | 0.026681 | yes |
| XLOC_006570 | HEATR6,gga-mir-6639 | 19:8668851-8684167 | 0.8% | 1.0% | 5.69613 | 86.0612 | 3.91731 | 9.93595 | 5.00E-05 | 0.00817 | yes |
| XLOC_006701 | DPP6 | 2:7411563-7635852 | 0.8% | 1.0% | 0.276706 | 8.75699 | 4.98401 | 6.1609 | 5.00E-05 | 0.00817 | yes |
| XLOC_006749 | ST8SIA6 | 2:19614632-19660208 | 0.8% | 1.0% | 1.85911 | 0.785796 | -1.24239 | -2.18858 | 0.00015 | 0.021491 | yes |
| XLOC_006926 | PDE1C | 2:48768262-49058756 | 0.8% | 1.0% | 6.04264 | 9.543 | 0.659263 | 2.09053 | 5.00E-05 | 0.00817 | yes |
| XLOC_006965 | COL15A1 | 2:55918315-56025683 | 0.8% | 1.0% | 2.6211 | 4.84035 | 0.88494 | 2.17763 | 5.00E-05 | 0.00817 | yes |
| XLOC_007017 | F13A1 | 2:65080950-65167267 | 0.8% | 1.0% | 32.0344 | 15.8481 | -1.01532 | -2.70084 | 5.00E-05 | 0.00817 | yes |
| XLOC_007043 | CDH10 | 2:72176696-72272302 | 0.8% | 1.0% | 9.55931 | 16.2233 | 0.763092 | 2.172 | 5.00E-05 | 0.00817 | yes |
| XLOC_007111 | ISG12(2) | 2:89722940-89729168 | 0.8% | 1.0% | 350.08 | 659.871 | 0.914498 | 2.84708 | 5.00E-05 | 0.00817 | yes |
| XLOC_007304 | DCSTAMP | 2:129726513-129736535 | 0.8% | 1.0% | 8.2834 | 3.14503 | -1.39715 | -1.9356 | 0.00015 | 0.021491 | yes |
| XLOC_007318 | NOV | 2:136064123-136069903 | 0.8% | 1.0% | 34.7477 | 53.6961 | 0.627902 | 1.98255 | 0.00025 | 0.031299 | yes |
| XLOC_007328 | FER1L6 | 2:137800983-137872216 | 0.8% | 1.0% | 11.3247 | 6.82523 | -0.73052 | -2.23467 | 0.00015 | 0.021491 | yes |
| XLOC_007355 | PSCA | 2:147858463-147863304 | 0.8% | 1.0% | 765.927 | 363.117 | -1.07677 | -3.42702 | 5.00E-05 | 0.00817 | yes |
| XLOC_007512 | ENSGALG00000009479 | 2:23037355-23046085 | 0.8% | 1.0% | 7.52058 | 18.2232 | 1.27686 | 3.5415 | 5.00E-05 | 0.00817 | yes |
| XLOC_007684 | TRANK1 | 2:46537409-46589650 | 0.8% | 1.0% | 18.7592 | 29.9271 | 0.673852 | 2.29695 | 5.00E-05 | 0.00817 | yes |
| XLOC_007896 | CCDC102B | 2:94086990-94213373 | 0.8% | 1.0% | 0.986791 | 2.80478 | 1.50707 | 1.81852 | 0.0003 | 0.036657 | yes |
| XLOC_008110 | SAMD12 | 2:135631890-135808926 | 0.8% | 1.0% | 1.27209 | 3.36981 | 1.40547 | 2.67247 | 5.00E-05 | 0.00817 | yes |
| XLOC_008112 | Enpp2 | 2:136104591-136163637 | 0.8% | 1.0% | 24.0158 | 43.7801 | 0.86629 | 2.90617 | 5.00E-05 | 0.00817 | yes |
| XLOC_008464 | - | 20:5515080-5515913 | 0.8% | 1.0% | 152.678 | 77.9726 | -0.96946 | -2.64537 | 5.00E-05 | 0.00817 | yes |
| XLOC_008607 | HELZ2 | 20:14255393-14279970 | 0.8% | 1.0% | 10.2008 | 17.1801 | 0.75206 | 2.5094 | 5.00E-05 | 0.00817 | yes |
| XLOC_008702 | PPDPF | 20:9368434-9370742 | 0.8% | 1.0% | 182.711 | 103.565 | -0.81903 | -2.6744 | 5.00E-05 | 0.00817 | yes |
| XLOC_009296 | STMN1 | 23:3198452-3201919 | 0.8% | 1.0% | 109.365 | 185.603 | 0.763065 | 2.46222 | 5.00E-05 | 0.00817 | yes |
| XLOC_009314 | CSF3R | 23:3964892-3977491 | 0.8% | 1.0% | 33.7986 | 16.525 | -1.03231 | -2.38905 | 0.00015 | 0.021491 | yes |
| XLOC_009444 | MECR | 23:2702691-2708797 | 0.8% | 1.0% | 14.2992 | 6.16899 | -1.21282 | -2.27766 | 5.00E-05 | 0.00817 | yes |
| XLOC_009766 | ENSGALG00000024272 | 25:734440-740904 | 0.8% | 1.0% | 232.777 | 149.426 | -0.63952 | -1.96361 | 0.00025 | 0.031299 | yes |
| XLOC_009893 | - | 25:1517789-1521784 | 0.8% | 1.0% | 180.58 | 86.2615 | -1.06585 | -2.2355 | 5.00E-05 | 0.00817 | yes |
| XLOC_010187 | ENSGALG00000028489 | 26:4975987-4979187 | 0.8% | 1.0% | 15.4029 | 5.85551 | -1.39534 | -2.54796 | 5.00E-05 | 0.00817 | yes |
| XLOC_010576 | REXO1,gga-mir-1647 | 28:2177989-2211436 | 0.8% | 1.0% | 171.157 | 17.8675 | -3.25992 | -12.6629 | 5.00E-05 | 0.00817 | yes |
| XLOC_010673 | SLC1A6 | 28:697352-724475 | 0.8% | 1.0% | 8.26733 | 3.5762 | -1.20899 | -2.51744 | 5.00E-05 | 0.00817 | yes |
| XLOC_010711 | MYO1F | 28:1729219-1742574 | 0.8% | 1.0% | 17.7808 | 11.1585 | -0.67218 | -2.02103 | 0.00015 | 0.021491 | yes |
| XLOC_010712 | ADAMTS10,gga-mir-6615 | 28:1743701-1792093 | 0.8% | 1.0% | 23.1882 | 91.7942 | 1.98501 | 8.77086 | 5.00E-05 | 0.00817 | yes |
| XLOC_010771 | GILT | 28:3759500-3762593 | 0.8% | 1.0% | 34.4011 | 17.9533 | -0.9382 | -2.7551 | 5.00E-05 | 0.00817 | yes |
| XLOC_010797 | R3HDM4 | 28:4727461-4733410 | 0.8% | 1.0% | 67.1331 | 39.3207 | -0.77173 | -2.12676 | 5.00E-05 | 0.00817 | yes |
| XLOC_010868 | XDH | 3:4394626-4438268 | 0.8% | 1.0% | 3.37232 | 1.43389 | -1.2338 | -2.46488 | 5.00E-05 | 0.00817 | yes |
| XLOC_011055 | EIF2AK2 | 3:31346861-31365625 | 0.8% | 1.0% | 16.8966 | 29.1707 | 0.787785 | 2.01434 | 0.00015 | 0.021491 | yes |
| XLOC_011386 | GSTA3 | 3:87797941-87804665 | 0.8% | 1.0% | 38.405 | 20.8949 | -0.87814 | -2.12313 | 0.0002 | 0.026681 | yes |
| XLOC_011403 | RSAD2 | 3:94588918-94596921 | 0.8% | 1.0% | 18.954 | 52.6395 | 1.47365 | 2.96376 | 5.00E-05 | 0.00817 | yes |
| XLOC_011501 | ENSGALG00000016682 | 3:108320647-108333075 | 0.8% | 1.0% | 12.7044 | 4.36233 | -1.54216 | -2.92922 | 5.00E-05 | 0.00817 | yes |
| XLOC_011670 | CENPF | 3:20356019-20391564 | 0.8% | 1.0% | 4.20404 | 6.65611 | 0.662904 | 1.93212 | 0.00025 | 0.031299 | yes |
| XLOC_011677 | ATF3 | 3:21184006-21192379 | 0.8% | 1.0% | 22.2254 | 41.7076 | 0.908099 | 2.46629 | 5.00E-05 | 0.00817 | yes |
| XLOC_011811 | SMOC2 | 3:40938587-41074750 | 0.8% | 1.0% | 12.2291 | 21.9417 | 0.843356 | 2.35941 | 5.00E-05 | 0.00817 | yes |
| XLOC_011830 | FNDC1 | 3:45071688-45111718 | 0.8% | 1.0% | 5.41293 | 8.46811 | 0.64563 | 1.89587 | 0.00025 | 0.031299 | yes |
| XLOC_011903 | FABP7 | 3:60674293-60677712 | 0.8% | 1.0% | 10.3516 | 27.0667 | 1.38667 | 2.41685 | 0.0001 | 0.015354 | yes |
| XLOC_012054 | CMPK2 | 3:94575874-94586375 | 0.8% | 1.0% | 18.5907 | 40.3691 | 1.11867 | 3.49892 | 5.00E-05 | 0.00817 | yes |
| XLOC_012467 | IL2RG | 4:2367898-2370689 | 0.8% | 1.0% | 35.8809 | 21.6493 | -0.7289 | -1.94206 | 0.00035 | 0.040343 | yes |
| XLOC_012642 | MGST2 | 4:28831967-28841789 | 0.8% | 1.0% | 19.6521 | 6.86675 | -1.51698 | -2.10407 | 0.00045 | 0.049221 | yes |
| XLOC_012689 | PYURF | 4:34474710-34493159 | 0.8% | 1.0% | 28.3337 | 54.4248 | 0.941743 | 2.28391 | 5.00E-05 | 0.00817 | yes |
| XLOC_012810 | NDNF | 4:53598091-53623955 | 0.8% | 1.0% | 10.5636 | 20.1018 | 0.928226 | 2.55424 | 5.00E-05 | 0.00817 | yes |
| XLOC_012848 | TLR3 | 4:60924043-60939133 | 0.8% | 1.0% | 10.5494 | 16.9385 | 0.683148 | 1.97527 | 0.00045 | 0.049221 | yes |
| XLOC_013229 | ENSGALG00000009639 | 4:24427017-24468687 | 0.8% | 1.0% | 3.6916 | 9.24386 | 1.32425 | 3.27166 | 5.00E-05 | 0.00817 | yes |
| XLOC_013436 | CENPE | 4:60597387-60634912 | 0.8% | 1.0% | 3.72689 | 6.45168 | 0.791704 | 2.21719 | 5.00E-05 | 0.00817 | yes |
| XLOC_013754 | - | 4:86220523-86221301 | 0.8% | 1.0% | 0 | 1.89092 | inf | #NAME? | 5.00E-05 | 0.00817 | yes |
| XLOC_013784 | GIF,MRPL16 | 5:483328-491033 | 0.8% | 1.0% | 177.735 | 107 | -0.73212 | -2.31159 | 5.00E-05 | 0.00817 | yes |
| XLOC_013801 | CASC5 | 5:920666-949219 | 0.8% | 1.0% | 3.20231 | 5.3053 | 0.728323 | 1.93389 | 0.00035 | 0.040343 | yes |
| XLOC_013890 | LDHA | 5:11936793-11942792 | 0.8% | 1.0% | 175.425 | 111.787 | -0.6501 | -2.19472 | 5.00E-05 | 0.00817 | yes |
| XLOC_013942 | APLNR | 5:16355331-16356501 | 0.8% | 1.0% | 34.9309 | 19.3182 | -0.85454 | -2.13496 | 0.00025 | 0.031299 | yes |
| XLOC_014128 | FOS | 5:37501646-37503967 | 0.8% | 1.0% | 52.7464 | 92.1797 | 0.805377 | 2.45471 | 5.00E-05 | 0.00817 | yes |
| XLOC_014406 | TPH1 | 5:11837182-11849973 | 0.8% | 1.0% | 8.70104 | 18.9762 | 1.12494 | 2.68026 | 5.00E-05 | 0.00817 | yes |
| XLOC_014774 | RPS29 | 5:57479789-57480844 | 0.8% | 1.0% | 122.202 | 74.5855 | -0.7123 | -2.10449 | 0.00015 | 0.021491 | yes |
| XLOC_015062 | ENSGALG00000006384 | 6:18900868-18904674 | 0.8% | 1.0% | 104.895 | 307.295 | 1.55068 | 4.32229 | 5.00E-05 | 0.00817 | yes |
| XLOC_015176 | PLPP4 | 6:30002734-30109050 | 0.8% | 1.0% | 0.661637 | 2.05872 | 1.63764 | 2.13124 | 0.0002 | 0.026681 | yes |
| XLOC_015239 | - | 6:3939382-3950467 | 0.8% | 1.0% | 80.3521 | 45.3391 | -0.82558 | -2.79803 | 5.00E-05 | 0.00817 | yes |
| XLOC_015357 | CXCL12 | 6:18559507-18577315 | 0.8% | 1.0% | 31.0269 | 48.8851 | 0.655874 | 2.11572 | 0.0001 | 0.015354 | yes |
| XLOC_015493 | - | 6:32285724-32349697 | 0.8% | 1.0% | 33.1073 | 20.4827 | -0.69275 | -2.28647 | 5.00E-05 | 0.00817 | yes |
| XLOC_016058 | MUC13 | 7:27422610-27442038 | 0.8% | 1.0% | 21.8031 | 35.3033 | 0.695266 | 2.10397 | 0.00015 | 0.021491 | yes |
| XLOC_016186 | ASPM | 8:2593759-2625802 | 0.8% | 1.0% | 4.49395 | 7.58428 | 0.755029 | 2.27292 | 5.00E-05 | 0.00817 | yes |
| XLOC_017184 | FAM43A | 9:12291915-12293004 | 0.8% | 1.0% | 36.7265 | 21.889 | -0.74661 | -1.91987 | 0.0002 | 0.026681 | yes |
| XLOC_017414 | - | AADN03009405.1:2-1516 | 0.8% | 1.0% | 13.2954 | 32.9335 | 1.30863 | 2.93962 | 5.00E-05 | 0.00817 | yes |
| XLOC_017538 | - | AADN03010736.1:801-1218 | 0.8% | 1.0% | 15.3036 | 86.5866 | 2.50027 | 3.87974 | 5.00E-05 | 0.00817 | yes |
| XLOC_017601 | ENSGALG00000023973 | AADN03011039.1:4-396 | 0.8% | 1.0% | 167.489 | 321.629 | 0.94133 | 2.51196 | 5.00E-05 | 0.00817 | yes |
| XLOC_017636 | - | AADN03011485.1:1901-3634 | 0.8% | 1.0% | 40.844 | 67.9705 | 0.734785 | 2.15488 | 5.00E-05 | 0.00817 | yes |
| XLOC_017821 | ENSGALG00000025779 | AADN03013108.1:4098-48954 | 0.8% | 1.0% | 1.52964 | 3.01652 | 0.979687 | 2.13975 | 0.00015 | 0.021491 | yes |
| XLOC_017999 | - | AADN03015080.1:2-1117 | 0.8% | 1.0% | 7.83713 | 20.2182 | 1.36726 | 2.31852 | 0.0001 | 0.015354 | yes |
| XLOC_018020 | - | AADN03015345.1:246-1961 | 0.8% | 1.0% | 12.6109 | 4.75928 | -1.40586 | -2.42038 | 0.0002 | 0.026681 | yes |
| XLOC_018022 | - | AADN03015356.1:395-777 | 0.8% | 1.0% | 9.17882 | 0 | #NAME? | #NAME? | 5.00E-05 | 0.00817 | yes |
| XLOC_018156 | - | AADN03016686.1:1-1878 | 0.8% | 1.0% | 0.794238 | 4.39185 | 2.46719 | 2.73909 | 0.00015 | 0.021491 | yes |
| XLOC_018230 | HMGA1 | AADN03017453.1:74-1374 | 0.8% | 1.0% | 2.76011 | 114.677 | 5.3767 | 6.24989 | 5.00E-05 | 0.00817 | yes |
| XLOC_018304 | - | AADN03018054.1:3-1083 | 0.8% | 1.0% | 11.3075 | 29.0613 | 1.36181 | 2.60481 | 0.0001 | 0.015354 | yes |
| XLOC_018374 | FCGBP | AADN03018760.1:359-10879 | 0.8% | 1.0% | 13.8191 | 23.6079 | 0.772607 | 2.27577 | 5.00E-05 | 0.00817 | yes |
| XLOC_018390 | - | AADN03018914.1:30-1595 | 0.8% | 1.0% | 0.408078 | 7.02057 | 4.10467 | 3.60326 | 0.00045 | 0.049221 | yes |
| XLOC_018619 | ENSGALG00000027312 | AADN03021393.1:20-6474 | 0.8% | 1.0% | 3.47891 | 7.64203 | 1.13532 | 2.21197 | 5.00E-05 | 0.00817 | yes |
| XLOC_018623 | - | AADN03021414.1:765-1014 | 0.8% | 1.0% | 9.92708 | 0 | #NAME? | #NAME? | 5.00E-05 | 0.00817 | yes |
| XLOC_018694 | - | AADN03022215.1:33-1619 | 0.8% | 1.0% | 1.37816 | 11.2849 | 3.03358 | 4.21827 | 5.00E-05 | 0.00817 | yes |
| XLOC_018764 | - | AADN03022946.1:1299-1580 | 0.8% | 1.0% | 4.61287 | 0 | #NAME? | #NAME? | 5.00E-05 | 0.00817 | yes |
| XLOC_018765 | - | AADN03022956.1:376-767 | 0.8% | 1.0% | 0 | 4.44112 | inf | #NAME? | 5.00E-05 | 0.00817 | yes |
| XLOC_018770 | C3d | AADN03022998.1:20-17720 | 0.8% | 1.0% | 14.6828 | 7.29037 | -1.01006 | -2.81873 | 5.00E-05 | 0.00817 | yes |
| XLOC_018877 | - | AADN03024286.1:74-463 | 0.8% | 1.0% | 0 | 5.86287 | inf | #NAME? | 5.00E-05 | 0.00817 | yes |
| XLOC_019190 | ENSGALG00000024340,ENSGALG00000027445 | JH375207.1:6221-21254 | 0.8% | 1.0% | 5.24083 | 20.9695 | 2.00042 | 2.9307 | 5.00E-05 | 0.00817 | yes |
| XLOC_019258 | - | JH375350.1:85-4183 | 0.8% | 1.0% | 1.159 | 36.9025 | 4.99276 | 7.08137 | 5.00E-05 | 0.00817 | yes |
| XLOC_019299 | - | JH375466.1:6424-7512 | 0.8% | 1.0% | 18.2546 | 51.1768 | 1.48723 | 3.31656 | 5.00E-05 | 0.00817 | yes |
| XLOC_019302 | - | JH375469.1:1-1593 | 0.8% | 1.0% | 9.36005 | 30.9361 | 1.7247 | 3.618 | 5.00E-05 | 0.00817 | yes |
| XLOC_019429 | ENSGALG00000000194 | JH375607.1:9152-27107 | 0.8% | 1.0% | 1.34755 | 4.22537 | 1.64873 | 2.6649 | 5.00E-05 | 0.00817 | yes |
| XLOC_019640 | - | JH376011.1:2828-3246 | 0.8% | 1.0% | 0 | 1.44997 | inf | #NAME? | 5.00E-05 | 0.00817 | yes |
| XLOC_019807 | - | JH376310.1:7544-8814 | 0.8% | 1.0% | 5.82902 | 1.15696 | -2.33292 | -2.62539 | 0.0002 | 0.026681 | yes |
| XLOC_019883 | - | JH376380.1:101586-102738 | 0.8% | 1.0% | 2.34884 | 0 | #NAME? | #NAME? | 5.00E-05 | 0.00817 | yes |
| XLOC_019889 | 17.5 | JH376396.1:31019-39226 | 0.8% | 1.0% | 9.88186 | 42.7284 | 2.11234 | 3.78903 | 5.00E-05 | 0.00817 | yes |
| XLOC_020112 | Worthington | Z:9023590-9024768 | 0.8% | 1.0% | 219.57 | 49.0346 | -2.16281 | -5.57705 | 5.00E-05 | 0.00817 | yes |
| XLOC_020244 | - | Z:30996489-31000549 | 0.8% | 1.0% | 8.87549 | 4.48847 | -0.98361 | -2.41187 | 5.00E-05 | 0.00817 | yes |
| XLOC_020356 | - | Z:54999279-55043601 | 0.8% | 1.0% | 2.80547 | 5.72777 | 1.02973 | 2.0248 | 0.0001 | 0.015354 | yes |
| XLOC_020858 | SMC2 | Z:66288329-66312320 | 0.8% | 1.0% | 22.7359 | 33.8692 | 0.575002 | 1.87369 | 0.00045 | 0.049221 | yes |
| XLOC_000282 | APOLD1 | 1:48046727-48051925 | 0.8% | 1.2% | 86.0232 | 36.1128 | -1.25222 | -3.81625 | 5.00E-05 | 0.00817 | yes |
| XLOC_000371 | ENSGALG00000027122 | 1:54174608-54204367 | 0.8% | 1.2% | 0.768342 | 4.32256 | 2.49207 | 2.64636 | 0.0004 | 0.045085 | yes |
| XLOC_000439 | ENSGALG00000013057 | 1:62212512-62227128 | 0.8% | 1.2% | 13.432 | 33.3823 | 1.31341 | 4.05005 | 5.00E-05 | 0.00817 | yes |
| XLOC_000569 | C1S | 1:76859214-76868460 | 0.8% | 1.2% | 19.0772 | 33.7776 | 0.82422 | 2.54081 | 5.00E-05 | 0.00817 | yes |
| XLOC_000600 | POLQ | 1:78861462-78918451 | 0.8% | 1.2% | 1.97037 | 3.62066 | 0.877791 | 2.06898 | 0.00045 | 0.049221 | yes |
| XLOC_000761 | Mx | 1:108275627-108317944 | 0.8% | 1.2% | 23.8275 | 66.4521 | 1.47969 | 3.5228 | 5.00E-05 | 0.00817 | yes |
| XLOC_001062 | ATP8A2 | 1:175972297-176290437 | 0.8% | 1.2% | 2.23102 | 0.784771 | -1.50736 | -2.0452 | 0.00045 | 0.049221 | yes |
| XLOC_001175 | HPX | 1:193596424-193599483 | 0.8% | 1.2% | 27.0104 | 11.8768 | -1.18537 | -2.77043 | 5.00E-05 | 0.00817 | yes |
| XLOC_001661 | ZC3HAV1 | 1:71546271-71571605 | 0.8% | 1.2% | 19.24 | 34.756 | 0.853155 | 2.77787 | 5.00E-05 | 0.00817 | yes |
| XLOC_001690 | MFAP5 | 1:75420698-75444541 | 0.8% | 1.2% | 38.3903 | 66.9738 | 0.802854 | 2.74341 | 5.00E-05 | 0.00817 | yes |
| XLOC_001906 | SIK1 | 1:109234332-109247225 | 0.8% | 1.2% | 31.7387 | 20.891 | -0.60336 | -2.09872 | 0.0004 | 0.045085 | yes |
| XLOC_002133 | EPSTI1 | 1:165956867-166019336 | 0.8% | 1.2% | 26.7873 | 49.8785 | 0.896869 | 2.04384 | 0.00035 | 0.040343 | yes |
| XLOC_002611 | - | 1:153879687-153881865 | 0.8% | 1.2% | 1.92923 | 0 | #NAME? | #NAME? | 5.00E-05 | 0.00817 | yes |
| XLOC_002822 | SCARNA15 | 10:10562099-10565827 | 0.8% | 1.2% | 6.99737 | 16.7705 | 1.26104 | 2.63094 | 0.0002 | 0.026681 | yes |
| XLOC_002904 | KIF23 | 10:18828577-18846460 | 0.8% | 1.2% | 9.45519 | 17.2753 | 0.869534 | 2.42164 | 0.0001 | 0.015354 | yes |
| XLOC_003016 | CCNB2 | 10:6313606-6321651 | 0.8% | 1.2% | 15.4666 | 26.4108 | 0.77197 | 2.16253 | 5.00E-05 | 0.00817 | yes |
| XLOC_003332 | RGS9BP | 11:9386093-9390346 | 0.8% | 1.2% | 6.69932 | 3.32968 | -1.00863 | -2.31913 | 0.00035 | 0.040343 | yes |
| XLOC_003472 | ENSGALG00000002237 | 11:1354998-1388751 | 0.8% | 1.2% | 25.3823 | 9.89164 | -1.35954 | -2.33484 | 5.00E-05 | 0.00817 | yes |
| XLOC_003733 | FBLN2 | 12:5763941-5869529 | 0.8% | 1.2% | 22.7085 | 34.1908 | 0.590374 | 1.94541 | 0.00045 | 0.049221 | yes |
| XLOC_003918 | OAS*A | 12:3221939-3228494 | 0.8% | 1.2% | 42.5503 | 81.6952 | 0.94108 | 2.74739 | 5.00E-05 | 0.00817 | yes |
| XLOC_003937 | NUP210 | 12:5607157-5668293 | 0.8% | 1.2% | 6.57086 | 11.2334 | 0.773637 | 2.46347 | 5.00E-05 | 0.00817 | yes |
| XLOC_004261 | LECT2 | 13:14532792-14537759 | 0.8% | 1.2% | 91.9329 | 261.115 | 1.50603 | 4.93833 | 5.00E-05 | 0.00817 | yes |
| XLOC_004365 | - | 13:7697747-7705169 | 0.8% | 1.2% | 25.5474 | 42.286 | 0.727 | 2.34144 | 5.00E-05 | 0.00817 | yes |
| XLOC_004545 | NPTX2 | 14:1182355-1234466 | 0.8% | 1.2% | 6.16759 | 2.41056 | -1.35534 | -2.38047 | 0.0002 | 0.026681 | yes |
| XLOC_005176 | CIT | 15:9566063-9637965 | 0.8% | 1.2% | 1.6541 | 3.23938 | 0.969673 | 2.18885 | 0.00035 | 0.040343 | yes |
| XLOC_005490 | TAP2 | 16:63364-67622 | 0.8% | 1.2% | 34.6758 | 19.7976 | -0.8086 | -2.51178 | 5.00E-05 | 0.00817 | yes |
| XLOC_005601 | LRRC8A | 17:5305567-5310846 | 0.8% | 1.2% | 4.25121 | 0.621003 | -2.7752 | -3.33461 | 5.00E-05 | 0.00817 | yes |
| XLOC_005725 | PTGDS | 17:509027-511253 | 0.8% | 1.2% | 78.0361 | 39.668 | -0.97617 | -2.4382 | 5.00E-05 | 0.00817 | yes |
| XLOC_006076 | MGAT5B | 18:4138710-4184210 | 0.8% | 1.2% | 5.79409 | 11.4443 | 0.981974 | 2.48986 | 5.00E-05 | 0.00817 | yes |
| XLOC_006117 | KPNA2 | 18:6953317-6960662 | 0.8% | 1.2% | 23.0304 | 44.1095 | 0.937553 | 2.86974 | 5.00E-05 | 0.00817 | yes |
| XLOC_006425 | ENSGALG00000001004 | 19:410266-421656 | 0.8% | 1.2% | 1.62071 | 4.09675 | 1.33785 | 2.08126 | 0.0003 | 0.036657 | yes |
| XLOC_006438 | ENSGALG00000027989 | 19:838833-843856 | 0.8% | 1.2% | 20.5693 | 0.710727 | -4.85505 | -3.80259 | 5.00E-05 | 0.00817 | yes |
| XLOC_006460 | ENSGALG00000001885 | 19:4176615-4178279 | 0.8% | 1.2% | 84.9425 | 50.212 | -0.75846 | -2.22571 | 0.00015 | 0.021491 | yes |
| XLOC_006701 | DPP6 | 2:7411563-7635852 | 0.8% | 1.2% | 0.276706 | 2.258 | 3.02862 | 3.39653 | 5.00E-05 | 0.00817 | yes |
| XLOC_006749 | ST8SIA6 | 2:19614632-19660208 | 0.8% | 1.2% | 1.85911 | 0.40882 | -2.18508 | -3.07029 | 5.00E-05 | 0.00817 | yes |
| XLOC_006926 | PDE1C | 2:48768262-49058756 | 0.8% | 1.2% | 6.04264 | 9.63069 | 0.672461 | 2.08712 | 0.00025 | 0.031299 | yes |
| XLOC_007111 | ISG12(2) | 2:89722940-89729168 | 0.8% | 1.2% | 350.08 | 751.974 | 1.103 | 3.51185 | 5.00E-05 | 0.00817 | yes |
| XLOC_007355 | PSCA | 2:147858463-147863304 | 0.8% | 1.2% | 765.927 | 435.803 | -0.81353 | -2.6087 | 5.00E-05 | 0.00817 | yes |
| XLOC_007433 | NCAPG2 | 2:9417856-9457336 | 0.8% | 1.2% | 1.32733 | 4.00598 | 1.59363 | 2.47032 | 5.00E-05 | 0.00817 | yes |
| XLOC_007462 | GAD2 | 2:16147742-16180423 | 0.8% | 1.2% | 1.71126 | 3.86926 | 1.177 | 2.36787 | 0.00015 | 0.021491 | yes |
| XLOC_007468 | OTUD1 | 2:17237192-17240043 | 0.8% | 1.2% | 60.0492 | 39.8596 | -0.59122 | -1.94374 | 0.00035 | 0.040343 | yes |
| XLOC_007512 | ENSGALG00000009479 | 2:23037355-23046085 | 0.8% | 1.2% | 7.52058 | 20.6929 | 1.46022 | 4.22242 | 5.00E-05 | 0.00817 | yes |
| XLOC_007520 | PDK4 | 2:24033199-24043554 | 0.8% | 1.2% | 11.6327 | 4.4023 | -1.40186 | -3.56808 | 5.00E-05 | 0.00817 | yes |
| XLOC_007668 | KIF15 | 2:43768480-43804535 | 0.8% | 1.2% | 7.11305 | 13.0722 | 0.877964 | 2.13652 | 0.00025 | 0.031299 | yes |
| XLOC_007691 | ANLN | 2:47314674-47343642 | 0.8% | 1.2% | 7.20659 | 16.8258 | 1.22329 | 3.42243 | 5.00E-05 | 0.00817 | yes |
| XLOC_007795 | SERPINB10 | 2:67745614-67752057 | 0.8% | 1.2% | 26.63 | 43.5799 | 0.710608 | 2.12614 | 0.0003 | 0.036657 | yes |
| XLOC_007933 | NDC80 | 2:100833427-100852126 | 0.8% | 1.2% | 5.80204 | 10.654 | 0.876767 | 2.31724 | 0.0001 | 0.015354 | yes |
| XLOC_008108 | ENSGALG00000026222 | 2:135233742-135377756 | 0.8% | 1.2% | 4.12191 | 15.4401 | 1.9053 | 2.84654 | 5.00E-05 | 0.00817 | yes |
| XLOC_008464 | - | 20:5515080-5515913 | 0.8% | 1.2% | 152.678 | 86.2887 | -0.82325 | -2.29165 | 0.00015 | 0.021491 | yes |
| XLOC_008486 | TGM3 | 20:6992785-7004637 | 0.8% | 1.2% | 2.7266 | 7.3872 | 1.43792 | 2.58261 | 5.00E-05 | 0.00817 | yes |
| XLOC_008544 | TPX2 | 20:10378632-10392964 | 0.8% | 1.2% | 11.2154 | 20.7646 | 0.888643 | 2.52352 | 5.00E-05 | 0.00817 | yes |
| XLOC_008607 | HELZ2 | 20:14255393-14279970 | 0.8% | 1.2% | 10.2008 | 15.8402 | 0.634909 | 2.09989 | 0.00035 | 0.040343 | yes |
| XLOC_008702 | PPDPF | 20:9368434-9370742 | 0.8% | 1.2% | 182.711 | 107.796 | -0.76126 | -2.50166 | 5.00E-05 | 0.00817 | yes |
| XLOC_009217 | ADRB3,ENSGALG00000003142,ENSGALG00000021824 | 22:2230162-2240201 | 0.8% | 1.2% | 40.2232 | 24.7393 | -0.70122 | -2.17145 | 0.0001 | 0.015354 | yes |
| XLOC_009296 | STMN1 | 23:3198452-3201919 | 0.8% | 1.2% | 109.365 | 208.005 | 0.92746 | 3.02145 | 5.00E-05 | 0.00817 | yes |
| XLOC_009810 | MCL1 | 25:1510951-1516430 | 0.8% | 1.2% | 224.964 | 139.026 | -0.69434 | -2.25489 | 5.00E-05 | 0.00817 | yes |
| XLOC_009893 | - | 25:1517789-1521784 | 0.8% | 1.2% | 180.58 | 95.5385 | -0.91849 | -1.96683 | 0.00045 | 0.049221 | yes |
| XLOC_010050 | TSPO2 | 26:4636546-4641634 | 0.8% | 1.2% | 4.0323 | 10.5465 | 1.38709 | 2.56129 | 0.0001 | 0.015354 | yes |
| XLOC_010135 | PIGR | 26:2520031-2542722 | 0.8% | 1.2% | 5.31861 | 12.9491 | 1.28373 | 3.17062 | 5.00E-05 | 0.00817 | yes |
| XLOC_010187 | ENSGALG00000028489 | 26:4975987-4979187 | 0.8% | 1.2% | 15.4029 | 6.04279 | -1.34992 | -2.53293 | 5.00E-05 | 0.00817 | yes |
| XLOC_010352 | ENSGALG00000023818 | 27:4835369-4836174 | 0.8% | 1.2% | 21.312 | 54.6955 | 1.35976 | 2.74087 | 5.00E-05 | 0.00817 | yes |
| XLOC_010463 | TOP2A | 27:4416595-4434189 | 0.8% | 1.2% | 11.0324 | 18.5367 | 0.748638 | 2.29252 | 0.0002 | 0.026681 | yes |
| XLOC_010501 | BRCA1 | 27:5149479-5171060 | 0.8% | 1.2% | 3.51213 | 6.41749 | 0.869663 | 2.25855 | 5.00E-05 | 0.00817 | yes |
| XLOC_010576 | REXO1,gga-mir-1647 | 28:2177989-2211436 | 0.8% | 1.2% | 171.157 | 16.3005 | -3.39233 | -12.5814 | 5.00E-05 | 0.00817 | yes |
| XLOC_010671 | LMNB2 | 28:631010-661875 | 0.8% | 1.2% | 32.7052 | 48.8732 | 0.579524 | 1.92158 | 0.0003 | 0.036657 | yes |
| XLOC_010784 | KLF2 | 28:4146805-4149435 | 0.8% | 1.2% | 130.943 | 66.2254 | -0.98348 | -3.47518 | 5.00E-05 | 0.00817 | yes |
| XLOC_010791 | UHRF1 | 28:4593479-4613058 | 0.8% | 1.2% | 13.7614 | 22.6575 | 0.719363 | 2.16452 | 0.00035 | 0.040343 | yes |
| XLOC_010797 | R3HDM4 | 28:4727461-4733410 | 0.8% | 1.2% | 67.1331 | 30.2133 | -1.15184 | -3.1178 | 5.00E-05 | 0.00817 | yes |
| XLOC_010844 | BUB1 | 3:3037096-3057283 | 0.8% | 1.2% | 3.73511 | 6.95963 | 0.897859 | 2.22385 | 0.00015 | 0.021491 | yes |
| XLOC_011403 | RSAD2 | 3:94588918-94596921 | 0.8% | 1.2% | 18.954 | 45.4818 | 1.26279 | 2.66486 | 5.00E-05 | 0.00817 | yes |
| XLOC_011411 | RRM2 | 3:96087194-96092245 | 0.8% | 1.2% | 15.0521 | 26.316 | 0.805972 | 2.15794 | 0.0004 | 0.045085 | yes |
| XLOC_011494 | GAL7 | 3:107069348-107075495 | 0.8% | 1.2% | 63.8067 | 163.641 | 1.35875 | 3.33961 | 5.00E-05 | 0.00817 | yes |
| XLOC_011495 | GAL2 | 3:107079760-107082276 | 0.8% | 1.2% | 59.4654 | 158.398 | 1.41343 | 3.58516 | 5.00E-05 | 0.00817 | yes |
| XLOC_011501 | ENSGALG00000016682 | 3:108320647-108333075 | 0.8% | 1.2% | 12.7044 | 3.94308 | -1.68793 | -3.20756 | 5.00E-05 | 0.00817 | yes |
| XLOC_011670 | CENPF | 3:20356019-20391564 | 0.8% | 1.2% | 4.20404 | 8.63857 | 1.03902 | 3.08256 | 5.00E-05 | 0.00817 | yes |
| XLOC_011830 | FNDC1 | 3:45071688-45111718 | 0.8% | 1.2% | 5.41293 | 9.16887 | 0.760333 | 2.2471 | 5.00E-05 | 0.00817 | yes |
| XLOC_011894 | CENPW | 3:59060254-59067276 | 0.8% | 1.2% | 7.28247 | 16.3908 | 1.17038 | 2.29205 | 0.00015 | 0.021491 | yes |
| XLOC_012054 | CMPK2 | 3:94575874-94586375 | 0.8% | 1.2% | 18.5907 | 50.4933 | 1.44151 | 4.62343 | 5.00E-05 | 0.00817 | yes |
| XLOC_012146 | GAL1 | 3:107083699-107086150 | 0.8% | 1.2% | 64.2461 | 161.533 | 1.33015 | 3.48896 | 5.00E-05 | 0.00817 | yes |
| XLOC_012302 | - | 3:49569079-49572733 | 0.8% | 1.2% | 0.823737 | 2.80566 | 1.76809 | 2.46686 | 0.00035 | 0.040343 | yes |
| XLOC_012426 | KIF4A | 4:1248685-1265951 | 0.8% | 1.2% | 5.50404 | 10.1649 | 0.885029 | 2.33644 | 0.00025 | 0.031299 | yes |
| XLOC_012678 | PLK4 | 4:33788059-33803011 | 0.8% | 1.2% | 3.83406 | 8.36928 | 1.12623 | 2.47662 | 5.00E-05 | 0.00817 | yes |
| XLOC_012689 | PYURF | 4:34474710-34493159 | 0.8% | 1.2% | 28.3337 | 53.3655 | 0.913387 | 2.37414 | 0.0001 | 0.015354 | yes |
| XLOC_012810 | NDNF | 4:53598091-53623955 | 0.8% | 1.2% | 10.5636 | 17.9476 | 0.764688 | 2.05758 | 0.00035 | 0.040343 | yes |
| XLOC_012966 | SPON2 | 4:84441719-84468575 | 0.8% | 1.2% | 1.23855 | 3.00875 | 1.28051 | 2.31665 | 5.00E-05 | 0.00817 | yes |
| XLOC_013046 | DRP2 | 4:2034111-2044107 | 0.8% | 1.2% | 4.51067 | 1.93954 | -1.21762 | -2.43697 | 5.00E-05 | 0.00817 | yes |
| XLOC_013229 | ENSGALG00000009639 | 4:24427017-24468687 | 0.8% | 1.2% | 3.6916 | 8.89911 | 1.26942 | 3.33611 | 5.00E-05 | 0.00817 | yes |
| XLOC_013436 | CENPE | 4:60597387-60634912 | 0.8% | 1.2% | 3.72689 | 7.99034 | 1.10029 | 3.13505 | 5.00E-05 | 0.00817 | yes |
| XLOC_013517 | NCAPG | 4:75480026-75503309 | 0.8% | 1.2% | 7.7025 | 14.76 | 0.938298 | 2.61371 | 5.00E-05 | 0.00817 | yes |
| XLOC_013754 | - | 4:86220523-86221301 | 0.8% | 1.2% | 0 | 1.63335 | inf | #NAME? | 5.00E-05 | 0.00817 | yes |
| XLOC_013784 | GIF,MRPL16 | 5:483328-491033 | 0.8% | 1.2% | 177.735 | 107.797 | -0.72141 | -2.38336 | 5.00E-05 | 0.00817 | yes |
| XLOC_013801 | CASC5 | 5:920666-949219 | 0.8% | 1.2% | 3.20231 | 6.14424 | 0.940123 | 2.54282 | 5.00E-05 | 0.00817 | yes |
| XLOC_013942 | APLNR | 5:16355331-16356501 | 0.8% | 1.2% | 34.9309 | 18.8155 | -0.89258 | -2.18029 | 5.00E-05 | 0.00817 | yes |
| XLOC_013943 | INCENP | 5:16370273-16411563 | 0.8% | 1.2% | 34.7685 | 130.903 | 1.91265 | 2.8148 | 0.0001 | 0.015354 | yes |
| XLOC_013944 | - | 5:16417722-16419137 | 0.8% | 1.2% | 16.4455 | 106.058 | 2.68909 | 5.29009 | 5.00E-05 | 0.00817 | yes |
| XLOC_014011 | CHAC1 | 5:23730405-23732558 | 0.8% | 1.2% | 12.6615 | 5.62563 | -1.17036 | -2.43767 | 5.00E-05 | 0.00817 | yes |
| XLOC_014257 | DLGAP5 | 5:56132154-56154143 | 0.8% | 1.2% | 12.0393 | 24.2782 | 1.01191 | 2.82816 | 5.00E-05 | 0.00817 | yes |
| XLOC_014774 | RPS29 | 5:57479789-57480844 | 0.8% | 1.2% | 122.202 | 72.2866 | -0.75746 | -2.22408 | 0.00015 | 0.021491 | yes |
| XLOC_015062 | ENSGALG00000006384 | 6:18900868-18904674 | 0.8% | 1.2% | 104.895 | 308.922 | 1.5583 | 4.47636 | 5.00E-05 | 0.00817 | yes |
| XLOC_015153 | ADRB1 | 6:27189840-27193270 | 0.8% | 1.2% | 15.7861 | 7.8924 | -1.00012 | -2.40426 | 0.0002 | 0.026681 | yes |
| XLOC_015181 | ENSGALG00000023424 | 6:30850163-30865389 | 0.8% | 1.2% | 1.18244 | 6.79746 | 2.52322 | 3.28534 | 5.00E-05 | 0.00817 | yes |
| XLOC_015200 | MKI67 | 6:32889668-32910556 | 0.8% | 1.2% | 15.9959 | 28.922 | 0.854468 | 2.72076 | 5.00E-05 | 0.00817 | yes |
| XLOC_015239 | - | 6:3939382-3950467 | 0.8% | 1.2% | 80.3521 | 51.8986 | -0.63064 | -2.15113 | 5.00E-05 | 0.00817 | yes |
| XLOC_015263 | CDK1 | 6:8142504-8170948 | 0.8% | 1.2% | 30.2554 | 50.4942 | 0.738923 | 2.06694 | 0.00045 | 0.049221 | yes |
| XLOC_015493 | - | 6:32285724-32349697 | 0.8% | 1.2% | 33.1073 | 19.498 | -0.76382 | -2.501 | 5.00E-05 | 0.00817 | yes |
| XLOC_015791 | PARP9 | 7:27763967-27772565 | 0.8% | 1.2% | 20.0921 | 31.2972 | 0.639406 | 2.1486 | 0.0002 | 0.026681 | yes |
| XLOC_015871 | - | 7:4903313-4963247 | 0.8% | 1.2% | 15.9042 | 5.80391 | -1.45431 | -3.18756 | 5.00E-05 | 0.00817 | yes |
| XLOC_016186 | ASPM | 8:2593759-2625802 | 0.8% | 1.2% | 4.49395 | 9.34988 | 1.05697 | 3.22175 | 5.00E-05 | 0.00817 | yes |
| XLOC_016346 | KLF2 | 8:19315885-19320497 | 0.8% | 1.2% | 33.9667 | 20.4097 | -0.73487 | -2.36449 | 5.00E-05 | 0.00817 | yes |
| XLOC_016524 | NUF2 | 8:5436611-5450983 | 0.8% | 1.2% | 6.55407 | 15.4749 | 1.23947 | 2.55571 | 5.00E-05 | 0.00817 | yes |
| XLOC_016662 | Rd | 8:20142536-20154566 | 0.8% | 1.2% | 19.3909 | 8.24116 | -1.23446 | -2.32493 | 5.00E-05 | 0.00817 | yes |
| XLOC_016843 | SLCO2A1 | 9:4063858-4088573 | 0.8% | 1.2% | 51.3592 | 33.5378 | -0.61483 | -2.03899 | 0.00025 | 0.031299 | yes |
| XLOC_017440 | - | AADN03009794.1:13248-19745 | 0.8% | 1.2% | 1.29549 | 0.225705 | -2.52098 | -2.37527 | 5.00E-05 | 0.00817 | yes |
| XLOC_017442 | - | AADN03009794.1:5311-8781 | 0.8% | 1.2% | 2.41547 | 0 | #NAME? | #NAME? | 5.00E-05 | 0.00817 | yes |
| XLOC_017526 | ENSGALG00000028245 | AADN03010684.1:2141-7309 | 0.8% | 1.2% | 14.6015 | 4.40596 | -1.72859 | -2.60736 | 5.00E-05 | 0.00817 | yes |
| XLOC_017555 | ENSGALG00000029041 | AADN03010818.1:4206-6452 | 0.8% | 1.2% | 39.4688 | 24.611 | -0.68141 | -2.04878 | 0.0003 | 0.036657 | yes |
| XLOC_017660 | - | AADN03011685.1:287-1112 | 0.8% | 1.2% | 0 | 1.26063 | inf | #NAME? | 5.00E-05 | 0.00817 | yes |
| XLOC_017779 | - | AADN03012812.1:770-1331 | 0.8% | 1.2% | 17.9955 | 4.35092 | -2.04825 | -2.56132 | 5.00E-05 | 0.00817 | yes |
| XLOC_018022 | - | AADN03015356.1:395-777 | 0.8% | 1.2% | 9.17882 | 0 | #NAME? | #NAME? | 5.00E-05 | 0.00817 | yes |
| XLOC_018220 | - | AADN03017366.1:45-1119 | 0.8% | 1.2% | 0 | 1.50372 | inf | #NAME? | 5.00E-05 | 0.00817 | yes |
| XLOC_018619 | ENSGALG00000027312 | AADN03021393.1:20-6474 | 0.8% | 1.2% | 3.47891 | 7.84355 | 1.17287 | 2.28667 | 0.00015 | 0.021491 | yes |
| XLOC_018623 | - | AADN03021414.1:765-1014 | 0.8% | 1.2% | 9.92708 | 0 | #NAME? | #NAME? | 5.00E-05 | 0.00817 | yes |
| XLOC_018624 | - | AADN03021418.1:0-1014 | 0.8% | 1.2% | 20.8656 | 4.04012 | -2.36866 | -3.02568 | 5.00E-05 | 0.00817 | yes |
| XLOC_018757 | - | AADN03022843.1:8737-11256 | 0.8% | 1.2% | 0.468869 | 2.61419 | 2.47911 | 2.6055 | 0.00025 | 0.031299 | yes |
| XLOC_018764 | - | AADN03022946.1:1299-1580 | 0.8% | 1.2% | 4.61287 | 0 | #NAME? | #NAME? | 0.00035 | 0.040343 | yes |
| XLOC_018795 | CDCA8 | AADN03023297.1:97-4363 | 0.8% | 1.2% | 32.0653 | 59.724 | 0.897297 | 2.26713 | 0.0001 | 0.015354 | yes |
| XLOC_018883 | - | AADN03024344.1:2191-2815 | 0.8% | 1.2% | 1.47893 | 0 | #NAME? | #NAME? | 5.00E-05 | 0.00817 | yes |
| XLOC_018913 | - | AADN03024630.1:6-1005 | 0.8% | 1.2% | 0.837467 | 9.88199 | 3.5607 | 3.58974 | 5.00E-05 | 0.00817 | yes |
| XLOC_018939 | ENSGALG00000018401 | AADN03024964.1:517-1148 | 0.8% | 1.2% | 0 | 28.9428 | inf | #NAME? | 5.00E-05 | 0.00817 | yes |
| XLOC_019074 | - | AADN03026566.1:5-1439 | 0.8% | 1.2% | 0.86953 | 5.21837 | 2.58529 | 2.7572 | 0.00035 | 0.040343 | yes |
| XLOC_019258 | - | JH375350.1:85-4183 | 0.8% | 1.2% | 1.159 | 26.3224 | 4.50533 | 6.16372 | 5.00E-05 | 0.00817 | yes |
| XLOC_019277 | - | JH375441.1:3148-3702 | 0.8% | 1.2% | 0 | 1.51366 | inf | #NAME? | 0.0002 | 0.026681 | yes |
| XLOC_019325 | - | JH375503.1:5493-6049 | 0.8% | 1.2% | 0 | 1.68344 | inf | #NAME? | 5.00E-05 | 0.00817 | yes |
| XLOC_019640 | - | JH376011.1:2828-3246 | 0.8% | 1.2% | 0 | 2.75728 | inf | #NAME? | 5.00E-05 | 0.00817 | yes |
| XLOC_019883 | - | JH376380.1:101586-102738 | 0.8% | 1.2% | 2.34884 | 0 | #NAME? | #NAME? | 5.00E-05 | 0.00817 | yes |
| XLOC_019889 | 17.5 | JH376396.1:31019-39226 | 0.8% | 1.2% | 9.88186 | 32.6365 | 1.72363 | 2.92553 | 5.00E-05 | 0.00817 | yes |
| XLOC_019909 | - | JH376407.1:34815-41168 | 0.8% | 1.2% | 1.28958 | 0.358036 | -1.84872 | -2.45874 | 0.0004 | 0.045085 | yes |
| XLOC_019920 | RACGAP1 | LGE22C19W28_E50C23:335215-343228 | 0.8% | 1.2% | 8.40938 | 16.2256 | 0.948204 | 2.29765 | 0.00015 | 0.021491 | yes |
| XLOC_020482 | ALPK2 | Z:767426-807344 | 0.8% | 1.2% | 2.64181 | 6.88178 | 1.38125 | 2.05373 | 0.00025 | 0.031299 | yes |
| XLOC_020803 | LPL | Z:54351097-54367889 | 0.8% | 1.2% | 23.3125 | 13.8859 | -0.74748 | -2.28039 | 5.00E-05 | 0.00817 | yes |
| XLOC_020858 | SMC2 | Z:66288329-66312320 | 0.8% | 1.2% | 22.7359 | 45.8676 | 1.0125 | 3.30638 | 5.00E-05 | 0.00817 | yes |
| XLOC_000446 | MGST1 | 1:63429469-63436121 | 1.0% | 1.2% | 61.5065 | 98.9543 | 0.686024 | 2.11509 | 5.00E-05 | 0.00817 | yes |
| XLOC_001062 | ATP8A2 | 1:175972297-176290437 | 1.0% | 1.2% | 2.24965 | 0.784771 | -1.51936 | -1.92141 | 0.0004 | 0.045085 | yes |
| XLOC_001189 | NUP98 | 1:193867709-193906260 | 1.0% | 1.2% | 42.2056 | 28.0539 | -0.58923 | -1.95191 | 0.00035 | 0.040343 | yes |
| XLOC_001286 | RELN | 1:12856182-13129087 | 1.0% | 1.2% | 13.7875 | 8.25621 | -0.73981 | -2.4272 | 5.00E-05 | 0.00817 | yes |
| XLOC_001694 | ENSGALG00000011930 | 1:75816668-75854119 | 1.0% | 1.2% | 4.57612 | 10.6236 | 1.21508 | 1.89375 | 0.00035 | 0.040343 | yes |
| XLOC_001761 | UPK1B | 1:80215124-80227530 | 1.0% | 1.2% | 5.61674 | 11.9371 | 1.08764 | 2.10869 | 0.00025 | 0.031299 | yes |
| XLOC_001859 | - | 1:103399230-103409113 | 1.0% | 1.2% | 5.05946 | 2.05145 | -1.30234 | -2.80983 | 5.00E-05 | 0.00817 | yes |
| XLOC_001899 | ENSGALG00000016165 | 1:108710397-108714893 | 1.0% | 1.2% | 43.364 | 88.1617 | 1.02366 | 2.12488 | 5.00E-05 | 0.00817 | yes |
| XLOC_001928 | Nox2 | 1:112488845-112516983 | 1.0% | 1.2% | 13.6242 | 28.7718 | 1.07848 | 2.62444 | 5.00E-05 | 0.00817 | yes |
| XLOC_001975 | TLR7 | 1:122843457-122863338 | 1.0% | 1.2% | 7.84974 | 16.8065 | 1.0983 | 2.63097 | 5.00E-05 | 0.00817 | yes |
| XLOC_002067 | - | 1:140710889-140714777 | 1.0% | 1.2% | 2.3488 | 8.56647 | 1.86677 | 2.17501 | 5.00E-05 | 0.00817 | yes |
| XLOC_002099 | - | 1:149163256-149164982 | 1.0% | 1.2% | 6.6898 | 13.628 | 1.02654 | 2.15493 | 5.00E-05 | 0.00817 | yes |
| XLOC_002262 | NAALAD2 | 1:186813719-186845023 | 1.0% | 1.2% | 5.86239 | 2.47653 | -1.24317 | -2.27993 | 0.0001 | 0.015354 | yes |
| XLOC_002268 | PRCP | 1:188219968-188248663 | 1.0% | 1.2% | 15.9995 | 25.2699 | 0.659393 | 2.09542 | 0.0002 | 0.026681 | yes |
| XLOC_002822 | SCARNA15 | 10:10562099-10565827 | 1.0% | 1.2% | 7.1744 | 16.7705 | 1.22499 | 2.5979 | 0.0001 | 0.015354 | yes |
| XLOC_004261 | LECT2 | 13:14532792-14537759 | 1.0% | 1.2% | 101.394 | 261.115 | 1.36472 | 4.41497 | 5.00E-05 | 0.00817 | yes |
| XLOC_004307 | EGR1 | 13:17704809-17707751 | 1.0% | 1.2% | 227.376 | 123.029 | -0.88608 | -2.71294 | 5.00E-05 | 0.00817 | yes |
| XLOC_004345 | DOCK2 | 13:3331843-3492674 | 1.0% | 1.2% | 15.9614 | 30.2522 | 0.922447 | 2.42199 | 5.00E-05 | 0.00817 | yes |
| XLOC_004353 | GABRA1 | 13:6492603-6646067 | 1.0% | 1.2% | 5.32276 | 1.44086 | -1.88525 | -3.24431 | 5.00E-05 | 0.00817 | yes |
| XLOC_005330 | ENSGALG00000021139 | 15:7928723-7935907 | 1.0% | 1.2% | 137.292 | 290.68 | 1.08219 | 3.055 | 5.00E-05 | 0.00817 | yes |
| XLOC_005723 | Ex-FABP | 17:501841-504845 | 1.0% | 1.2% | 357.186 | 924.468 | 1.37195 | 3.85817 | 5.00E-05 | 0.00817 | yes |
| XLOC_005790 | SH3GLB2 | 17:5432743-5457800 | 1.0% | 1.2% | 4.54423 | 0.653887 | -2.79692 | -3.54158 | 5.00E-05 | 0.00817 | yes |
| XLOC_006076 | MGAT5B | 18:4138710-4184210 | 1.0% | 1.2% | 5.94414 | 11.4443 | 0.945088 | 2.55889 | 5.00E-05 | 0.00817 | yes |
| XLOC_006305 | RABGEF1,TMEM248,gga-mir-6585 | 19:5031339-5068523 | 1.0% | 1.2% | 151.718 | 87.0524 | -0.80143 | -3.11147 | 5.00E-05 | 0.00817 | yes |
| XLOC_006425 | ENSGALG00000001004 | 19:410266-421656 | 1.0% | 1.2% | 1.18987 | 4.09675 | 1.78367 | 2.61857 | 5.00E-05 | 0.00817 | yes |
| XLOC_006438 | ENSGALG00000027989 | 19:838833-843856 | 1.0% | 1.2% | 19.788 | 0.710727 | -4.79919 | -3.67923 | 5.00E-05 | 0.00817 | yes |
| XLOC_006570 | HEATR6,gga-mir-6639 | 19:8668851-8684167 | 1.0% | 1.2% | 86.0612 | 5.77354 | -3.89783 | -9.97655 | 5.00E-05 | 0.00817 | yes |
| XLOC_006701 | DPP6 | 2:7411563-7635852 | 1.0% | 1.2% | 8.75699 | 2.258 | -1.95539 | -3.82904 | 5.00E-05 | 0.00817 | yes |
| XLOC_006733 | ARMC4 | 2:15517574-15589648 | 1.0% | 1.2% | 0.569502 | 1.80241 | 1.66216 | 1.94864 | 0.00035 | 0.040343 | yes |
| XLOC_007017 | F13A1 | 2:65080950-65167267 | 1.0% | 1.2% | 15.8481 | 29.0917 | 0.876301 | 2.31866 | 5.00E-05 | 0.00817 | yes |
| XLOC_007304 | DCSTAMP | 2:129726513-129736535 | 1.0% | 1.2% | 3.14503 | 10.8334 | 1.78434 | 2.51384 | 5.00E-05 | 0.00817 | yes |
| XLOC_007318 | NOV | 2:136064123-136069903 | 1.0% | 1.2% | 53.6961 | 32.7487 | -0.71338 | -2.19097 | 5.00E-05 | 0.00817 | yes |
| XLOC_007403 | CATHL1,CATHL2 | 2:3982184-3987898 | 1.0% | 1.2% | 20.6447 | 49.107 | 1.25016 | 2.35044 | 0.0001 | 0.015354 | yes |
| XLOC_007462 | GAD2 | 2:16147742-16180423 | 1.0% | 1.2% | 2.04598 | 3.86926 | 0.919262 | 1.88839 | 0.00045 | 0.049221 | yes |
| XLOC_007468 | OTUD1 | 2:17237192-17240043 | 1.0% | 1.2% | 60.9189 | 39.8596 | -0.61196 | -1.99531 | 0.00025 | 0.031299 | yes |
| XLOC_007488 | ENSGALG00000028304 | 2:19421327-19450406 | 1.0% | 1.2% | 12.0671 | 19.4046 | 0.685314 | 2.2463 | 5.00E-05 | 0.00817 | yes |
| XLOC_007520 | PDK4 | 2:24033199-24043554 | 1.0% | 1.2% | 7.98522 | 4.4023 | -0.85907 | -2.13761 | 0.00025 | 0.031299 | yes |
| XLOC_007621 | cTR | 2:37839380-38004659 | 1.0% | 1.2% | 13.6468 | 7.82298 | -0.80277 | -2.16886 | 0.00025 | 0.031299 | yes |
| XLOC_007775 | LY86 | 2:64976837-65001998 | 1.0% | 1.2% | 51.3661 | 85.423 | 0.733808 | 2.44568 | 5.00E-05 | 0.00817 | yes |
| XLOC_007866 | ENSGALG00000013268 | 2:88761815-88910242 | 1.0% | 1.2% | 0.456489 | 1.19276 | 1.38566 | 2.50645 | 5.00E-05 | 0.00817 | yes |
| XLOC_008110 | SAMD12 | 2:135631890-135808926 | 1.0% | 1.2% | 3.36981 | 1.43858 | -1.22802 | -2.45492 | 5.00E-05 | 0.00817 | yes |
| XLOC_008112 | Enpp2 | 2:136104591-136163637 | 1.0% | 1.2% | 43.7801 | 27.6522 | -0.66288 | -2.24614 | 5.00E-05 | 0.00817 | yes |
| XLOC_008486 | TGM3 | 20:6992785-7004637 | 1.0% | 1.2% | 3.39304 | 7.3872 | 1.12245 | 2.12938 | 0.00035 | 0.040343 | yes |
| XLOC_009195 | PRNP | 22:430810-436437 | 1.0% | 1.2% | 8.84715 | 15.1788 | 0.778776 | 1.92348 | 0.00045 | 0.049221 | yes |
| XLOC_009766 | ENSGALG00000024272 | 25:734440-740904 | 1.0% | 1.2% | 149.426 | 237.462 | 0.668267 | 2.05167 | 5.00E-05 | 0.00817 | yes |
| XLOC_010135 | PIGR | 26:2520031-2542722 | 1.0% | 1.2% | 4.63539 | 12.9491 | 1.48209 | 3.48266 | 5.00E-05 | 0.00817 | yes |
| XLOC_010363 | WNK4 | 27:5039149-5053606 | 1.0% | 1.2% | 14.6028 | 9.24995 | -0.65873 | -1.88083 | 0.0004 | 0.045085 | yes |
| XLOC_010673 | SLC1A6 | 28:697352-724475 | 1.0% | 1.2% | 3.5762 | 11.1215 | 1.63685 | 3.42821 | 5.00E-05 | 0.00817 | yes |
| XLOC_010712 | ADAMTS10,gga-mir-6615 | 28:1743701-1792093 | 1.0% | 1.2% | 91.7942 | 20.0038 | -2.19813 | -9.35925 | 5.00E-05 | 0.00817 | yes |
| XLOC_010784 | KLF2 | 28:4146805-4149435 | 1.0% | 1.2% | 104.318 | 66.2254 | -0.65553 | -2.29482 | 5.00E-05 | 0.00817 | yes |
| XLOC_010868 | XDH | 3:4394626-4438268 | 1.0% | 1.2% | 1.43389 | 3.62962 | 1.33988 | 2.7478 | 5.00E-05 | 0.00817 | yes |
| XLOC_011127 | RNASET2 | 3:41810075-41832795 | 1.0% | 1.2% | 21.7954 | 35.1661 | 0.690159 | 1.94079 | 0.00035 | 0.040343 | yes |
| XLOC_011203 | IL22RA2 | 3:54113756-54119427 | 1.0% | 1.2% | 1.23985 | 3.93806 | 1.66732 | 2.13615 | 0.0002 | 0.026681 | yes |
| XLOC_011386 | GSTA3 | 3:87797941-87804665 | 1.0% | 1.2% | 20.8949 | 45.1721 | 1.11228 | 2.68363 | 5.00E-05 | 0.00817 | yes |
| XLOC_011387 | ENSGALG00000028551 | 3:87804919-87828658 | 1.0% | 1.2% | 32.3553 | 53.6327 | 0.729113 | 2.06507 | 0.0002 | 0.026681 | yes |
| XLOC_011494 | GAL7 | 3:107069348-107075495 | 1.0% | 1.2% | 62.0047 | 163.641 | 1.40008 | 3.42798 | 5.00E-05 | 0.00817 | yes |
| XLOC_011495 | GAL2 | 3:107079760-107082276 | 1.0% | 1.2% | 66.455 | 158.398 | 1.2531 | 3.26923 | 5.00E-05 | 0.00817 | yes |
| XLOC_011811 | SMOC2 | 3:40938587-41074750 | 1.0% | 1.2% | 21.9417 | 12.0287 | -0.8672 | -2.44504 | 5.00E-05 | 0.00817 | yes |
| XLOC_011997 | - | 3:79589116-79607036 | 1.0% | 1.2% | 0.540939 | 1.64895 | 1.60801 | 2.20376 | 0.0002 | 0.026681 | yes |
| XLOC_012113 | ENSGALG00000016556 | 3:104624629-104639398 | 1.0% | 1.2% | 7.09851 | 12.4379 | 0.809157 | 2.47445 | 5.00E-05 | 0.00817 | yes |
| XLOC_012146 | GAL1 | 3:107083699-107086150 | 1.0% | 1.2% | 57.8385 | 161.533 | 1.48172 | 3.8044 | 5.00E-05 | 0.00817 | yes |
| XLOC_013228 | SPOCK3 | 4:23945606-24110706 | 1.0% | 1.2% | 8.18694 | 15.7312 | 0.942233 | 2.01977 | 0.0003 | 0.036657 | yes |
| XLOC_013890 | LDHA | 5:11936793-11942792 | 1.0% | 1.2% | 111.787 | 215.185 | 0.944828 | 3.07252 | 5.00E-05 | 0.00817 | yes |
| XLOC_013944 | - | 5:16417722-16419137 | 1.0% | 1.2% | 35.6087 | 106.058 | 1.57455 | 3.34199 | 5.00E-05 | 0.00817 | yes |
| XLOC_013991 | SPI1 | 5:22247549-22268506 | 1.0% | 1.2% | 51.7029 | 82.6141 | 0.676143 | 2.08449 | 0.0002 | 0.026681 | yes |
| XLOC_014011 | CHAC1 | 5:23730405-23732558 | 1.0% | 1.2% | 19.3365 | 5.62563 | -1.78124 | -3.73854 | 5.00E-05 | 0.00817 | yes |
| XLOC_014027 | CAPN3 | 5:24937024-24961725 | 1.0% | 1.2% | 0.811221 | 2.50738 | 1.62801 | 2.28525 | 5.00E-05 | 0.00817 | yes |
| XLOC_014128 | FOS | 5:37501646-37503967 | 1.0% | 1.2% | 92.1797 | 54.852 | -0.7489 | -2.34312 | 5.00E-05 | 0.00817 | yes |
| XLOC_015151 | PLEKHS1 | 6:27064758-27081909 | 1.0% | 1.2% | 1.93935 | 3.94648 | 1.025 | 1.98131 | 0.00035 | 0.040343 | yes |
| XLOC_015181 | ENSGALG00000023424 | 6:30850163-30865389 | 1.0% | 1.2% | 1.10966 | 6.79746 | 2.61488 | 3.55683 | 5.00E-05 | 0.00817 | yes |
| XLOC_015871 | - | 7:4903313-4963247 | 1.0% | 1.2% | 12.0425 | 5.80391 | -1.05303 | -2.42323 | 5.00E-05 | 0.00817 | yes |
| XLOC_016662 | Rd | 8:20142536-20154566 | 1.0% | 1.2% | 32.9853 | 8.24116 | -2.0009 | -3.84936 | 5.00E-05 | 0.00817 | yes |
| XLOC_016903 | CCL20 | 9:9020463-9022561 | 1.0% | 1.2% | 11.9886 | 30.3687 | 1.34092 | 2.62586 | 5.00E-05 | 0.00817 | yes |
| XLOC_017111 | TF | 9:4096868-4107617 | 1.0% | 1.2% | 53.7326 | 105.121 | 0.968186 | 3.40319 | 5.00E-05 | 0.00817 | yes |
| XLOC_017440 | - | AADN03009794.1:13248-19745 | 1.0% | 1.2% | 1.32861 | 0.225705 | -2.55741 | -2.41593 | 5.00E-05 | 0.00817 | yes |
| XLOC_017441 | - | AADN03009794.1:14-5229 | 1.0% | 1.2% | 1.50913 | 0 | #NAME? | #NAME? | 5.00E-05 | 0.00817 | yes |
| XLOC_017442 | - | AADN03009794.1:5311-8781 | 1.0% | 1.2% | 3.53568 | 0 | #NAME? | #NAME? | 5.00E-05 | 0.00817 | yes |
| XLOC_017526 | ENSGALG00000028245 | AADN03010684.1:2141-7309 | 1.0% | 1.2% | 13.2069 | 4.40596 | -1.58376 | -1.99426 | 5.00E-05 | 0.00817 | yes |
| XLOC_017538 | - | AADN03010736.1:801-1218 | 1.0% | 1.2% | 86.5866 | 31.3614 | -1.46515 | -2.61591 | 5.00E-05 | 0.00817 | yes |
| XLOC_017636 | - | AADN03011485.1:1901-3634 | 1.0% | 1.2% | 67.9705 | 43.7484 | -0.63568 | -1.88963 | 0.00035 | 0.040343 | yes |
| XLOC_017786 | - | AADN03012854.1:359-709 | 1.0% | 1.2% | 5.3195 | 0 | #NAME? | #NAME? | 5.00E-05 | 0.00817 | yes |
| XLOC_017821 | ENSGALG00000025779 | AADN03013108.1:4098-48954 | 1.0% | 1.2% | 3.01652 | 1.3999 | -1.10756 | -2.4066 | 5.00E-05 | 0.00817 | yes |
| XLOC_018156 | - | AADN03016686.1:1-1878 | 1.0% | 1.2% | 4.39185 | 0.794792 | -2.46618 | -2.81636 | 5.00E-05 | 0.00817 | yes |
| XLOC_018222 | - | AADN03017375.1:94-1322 | 1.0% | 1.2% | 32.8993 | 17.8265 | -0.88404 | -2.0907 | 0.00025 | 0.031299 | yes |
| XLOC_018230 | HMGA1 | AADN03017453.1:74-1374 | 1.0% | 1.2% | 114.677 | 2.89055 | -5.31008 | -6.30236 | 5.00E-05 | 0.00817 | yes |
| XLOC_018263 | - | AADN03017695.1:999-1464 | 1.0% | 1.2% | 0 | 1.93455 | inf | #NAME? | 5.00E-05 | 0.00817 | yes |
| XLOC_018374 | FCGBP | AADN03018760.1:359-10879 | 1.0% | 1.2% | 23.6079 | 12.086 | -0.96594 | -2.78157 | 5.00E-05 | 0.00817 | yes |
| XLOC_018519 | - | AADN03020228.1:5-744 | 1.0% | 1.2% | 11.5542 | 26.1278 | 1.17717 | 2.09007 | 0.00035 | 0.040343 | yes |
| XLOC_018529 | - | AADN03020342.1:447-1263 | 1.0% | 1.2% | 17.1808 | 35.6108 | 1.05151 | 2.17121 | 0.00025 | 0.031299 | yes |
| XLOC_018624 | - | AADN03021418.1:0-1014 | 1.0% | 1.2% | 13.9209 | 4.04012 | -1.78479 | -2.16138 | 0.0004 | 0.045085 | yes |
| XLOC_018694 | - | AADN03022215.1:33-1619 | 1.0% | 1.2% | 11.2849 | 1.50763 | -2.90404 | -4.0436 | 5.00E-05 | 0.00817 | yes |
| XLOC_018765 | - | AADN03022956.1:376-767 | 1.0% | 1.2% | 4.44112 | 0 | #NAME? | #NAME? | 5.00E-05 | 0.00817 | yes |
| XLOC_018795 | CDCA8 | AADN03023297.1:97-4363 | 1.0% | 1.2% | 34.6721 | 59.724 | 0.784535 | 2.01931 | 0.00025 | 0.031299 | yes |
| XLOC_018909 | - | AADN03024594.1:274-663 | 1.0% | 1.2% | 2.2572 | 0 | #NAME? | #NAME? | 0.0002 | 0.026681 | yes |
| XLOC_018913 | - | AADN03024630.1:6-1005 | 1.0% | 1.2% | 1.33898 | 9.88199 | 2.88367 | 3.07481 | 5.00E-05 | 0.00817 | yes |
| XLOC_019190 | ENSGALG00000024340,ENSGALG00000027445 | JH375207.1:6221-21254 | 1.0% | 1.2% | 20.9695 | 5.67805 | -1.88482 | -3.35267 | 5.00E-05 | 0.00817 | yes |
| XLOC_019429 | ENSGALG00000000194 | JH375607.1:9152-27107 | 1.0% | 1.2% | 4.22537 | 1.74457 | -1.27621 | -2.08486 | 0.0004 | 0.045085 | yes |
| XLOC_019990 | ENSGALG00000009103 | LGE64:606953-658685 | 1.0% | 1.2% | 20.9346 | 35.5158 | 0.76257 | 1.95129 | 0.0003 | 0.036657 | yes |
| XLOC_020112 | Worthington | Z:9023590-9024768 | 1.0% | 1.2% | 49.0346 | 201.719 | 2.04047 | 5.14096 | 5.00E-05 | 0.00817 | yes |
| XLOC_020244 | - | Z:30996489-31000549 | 1.0% | 1.2% | 4.48847 | 8.29081 | 0.885291 | 2.15041 | 5.00E-05 | 0.00817 | yes |
| XLOC_020624 | CD180 | Z:21157449-21166375 | 1.0% | 1.2% | 1.78061 | 4.72527 | 1.40802 | 2.22675 | 0.0003 | 0.036657 | yes |
| XLOC_020831 | VCAN | Z:62473942-62580859 | 1.0% | 1.2% | 11.8545 | 18.2209 | 0.620163 | 2.12799 | 5.00E-05 | 0.00817 | yes |
| XLOC_020991 | - | Z:47228187-47236741 | 1.0% | 1.2% | 5.11391 | 8.13689 | 0.67005 | 1.94703 | 5.00E-05 | 0.00817 | yes |

**Supplementary Table S3. Enriched KEGG pathways of the DEGs identified using the edgeR tool.**

| **Gene symbol** | **Gene full name** | **KEGG Pathway 1** | **KEGG Pathway 2** |
| --- | --- | --- | --- |
| *KLF2* | Krueppel-like factor 2 | FoxO signaling pathway | Embryonic and Induced Pluripotent Stem Cell Differentiation Pathways |
| *ADAMTS8* | ADAM metallopeptidase with thrombospondin type 1 motif, 8 | Degradation of the extracellular matrix | O-linked glycosylation |
| *AP3S2* | Adaptor related protein complex 3 sigma 2 subunit | Lysosome |  |
| *PGC* | Progastricsin (pepsinogen C) |  |  |
| *HPX* | Hemopexin |  |  |
| *FABP4* | Fatty acid-binding protein 4 | PPAR signaling pathway | Regulation of lipolysis in adipocytes |
| *LECT2* | Leukocyte cell derived chemotaxin 2 |  |  |
| *NUP210* | Nucleoporin 210kDa | RNA transport |  |
| *GAL2* | Galanin receptor 2 | Neuroactive ligand-receptor interaction |  |
| *TMEM8A* | Transmembrane protein 8A |  |  |
| ENSGALG00000028428 | Novel gene |  |  |
| ENSGALG00000028627 | Novel gene |  |  |

**Supplementary Table S4. Annotation of the co-occurrence of DEGs identified using the edgeR/cufflinks tools with IPAD database.**

| **Disease ID** | **Disease Name** | **Gene** | **AE** | **RE** | **N** | **MJI** | **P-value** |
| --- | --- | --- | --- | --- | --- | --- | --- |
| MESH:D056486 | Drug-Induced Liver Injury | PGC;LECT2;NUP210;HPX;FABP4;KLF2;ADAMTS8;AP3S2 | 8 | 1.17 | 15300 | 0.5003 | 0.691168 |
| MESH:D009336 | Necrosis | AP3S2;ADAMTS8;FABP4;KLF2;PGC;NUP210;LECT2;HPX | 8 | 1.21 | 14853 | 0.5003 | 0.691168 |
| MESH:D006973 | Hypertension | NUP210;LECT2;PGC;FABP4;HPX;ADAMTS8;KLF2;AP3S2 | 8 | 1.26 | 14280 | 0.5003 | 0.691168 |
| MESH:D004362 | Drug Toxicity | AP3S2;ADAMTS8;KLF2;FABP4;HPX;NUP210;LECT2;PGC | 8 | 1.27 | 14107 | 0.5003 | 0.691168 |
| MESH:D005234 | Fatty Liver | AP3S2;ADAMTS8;KLF2;HPX;FABP4;LECT2;PGC;NUP210 | 8 | 1.27 | 14162 | 0.5003 | 0.691168 |
| MESH:D008107 | Liver Diseases | FABP4;HPX;PGC;LECT2;NUP210;KLF2;ADAMTS8;AP3S2 | 8 | 1.27 | 14090 | 0.5003 | 0.691168 |
| MESH:D007674 | Kidney Diseases | FABP4;HPX;NUP210;PGC;LECT2;ADAMTS8;KLF2;AP3S2 | 8 | 1.28 | 14039 | 0.5003 | 0.691168 |
| MESH:D006965 | Hyperplasia | HPX;FABP4;LECT2;PGC;NUP210;KLF2;ADAMTS8;AP3S2 | 8 | 1.28 | 13955 | 0.5003 | 0.691168 |
| MESH:D010146 | Pain | KLF2;FABP4;ADAMTS8;AP3S2;LECT2;NUP210;PGC;HPX | 8 | 1.28 | 13977 | 0.5003 | 0.691168 |
| MESH:D002779 | Cholestasis | ADAMTS8;AP3S2;KLF2;NUP210;PGC;LECT2;FABP4;HPX | 8 | 1.3 | 13836 | 0.5003 | 0.691168 |
| MESH:D006528 | Carcinoma, Hepatocellular | ADAMTS8;AP3S2;KLF2;PGC;LECT2;NUP210;HPX;FABP4 | 8 | 1.3 | 13821 | 0.5003 | 0.691168 |
| MESH:D009325 | Nausea | HPX;LECT2;NUP210;PGC;KLF2;FABP4;ADAMTS8;AP3S2 | 8 | 1.3 | 13826 | 0.5003 | 0.691168 |
| MESH:D011230 | Precancerous Conditions | LECT2;PGC;HPX;NUP210;AP3S2;FABP4;KLF2;ADAMTS8 | 8 | 1.3 | 13753 | 0.5003 | 0.691168 |
| MESH:D006470 | Hemorrhage | KLF2;ADAMTS8;AP3S2;PGC;LECT2;NUP210;HPX;FABP4 | 8 | 1.32 | 13601 | 0.5003 | 0.691168 |
| MESH:D006261 | Headache | FABP4;HPX;PGC;LECT2;NUP210;KLF2;ADAMTS8;AP3S2 | 8 | 1.32 | 13561 | 0.5003 | 0.691168 |
| MESH:D004487 | Edema | FABP4;HPX;LECT2;PGC;NUP210;KLF2;AP3S2;ADAMTS8 | 8 | 1.33 | 13464 | 0.5003 | 0.691168 |
| MESH:D009422 | Nervous System Diseases | PGC;LECT2;NUP210;HPX;ADAMTS8;FABP4;KLF2;AP3S2 | 8 | 1.33 | 13526 | 0.5003 | 0.691168 |
| MESH:D001943 | Breast Neoplasms | AP3S2;ADAMTS8;KLF2;LECT2;PGC;NUP210;HPX;FABP4 | 8 | 1.34 | 13397 | 0.5003 | 0.691168 |
| MESH:D051437 | Renal Insufficiency | NUP210;HPX;PGC;LECT2;KLF2;FABP4;ADAMTS8;AP3S2 | 8 | 1.35 | 13241 | 0.5003 | 0.691168 |
| MESH:D007249 | Inflammation | KLF2;ADAMTS8;AP3S2;PGC;LECT2;NUP210;HPX;FABP4 | 8 | 1.35 | 13254 | 0.5003 | 0.691168 |
| MESH:D001927 | Brain Diseases | FABP4;HPX;LECT2;PGC;NUP210;ADAMTS8;AP3S2;KLF2 | 8 | 1.36 | 13134 | 0.5003 | 0.691168 |
| MESH:D003072 | Cognition Disorders | KLF2;ADAMTS8;AP3S2;NUP210;PGC;LECT2;FABP4;HPX | 8 | 1.37 | 13131 | 0.5003 | 0.691168 |
| MESH:D011507 | Proteinuria | KLF2;FABP4;ADAMTS8;AP3S2;HPX;NUP210;PGC;LECT2 | 8 | 1.37 | 13045 | 0.5003 | 0.691168 |
| MESH:D005334 | Fever | NUP210;LECT2;PGC;FABP4;HPX;KLF2;ADAMTS8;AP3S2 | 8 | 1.39 | 12889 | 0.5003 | 0.691168 |
| MESH:D009369 | Neoplasms | FABP4;ADAMTS8;KLF2;AP3S2;HPX;PGC;NUP210;LECT2 | 8 | 1.39 | 12925 | 0.5003 | 0.691168 |
| MESH:D000743 | Anemia, Hemolytic | HPX;ADAMTS8;AP3S2;LECT2;PGC;NUP210;KLF2;FABP4 | 8 | 1.4 | 12764 | 0.5003 | 0.691168 |
| MESH:D013375 | Substance Withdrawal Syndrome | FABP4;ADAMTS8;KLF2;AP3S2;NUP210;HPX;LECT2;PGC | 8 | 1.42 | 12665 | 0.5003 | 0.691168 |
| MESH:D000230 | Adenocarcinoma | NUP210;PGC;LECT2;FABP4;KLF2;HPX;AP3S2;ADAMTS8 | 8 | 1.43 | 12525 | 0.5003 | 0.691168 |
| MESH:D058186 | Acute Kidney Injury | NUP210;PGC;FABP4;LECT2;KLF2;HPX;ADAMTS8;AP3S2 | 8 | 1.43 | 12527 | 0.5003 | 0.691168 |
| MESH:D003866 | Depressive Disorder | FABP4;HPX;PGC;LECT2;NUP210;KLF2;ADAMTS8;AP3S2 | 8 | 1.43 | 12565 | 0.5003 | 0.691168 |
| MESH:D007680 | Kidney Neoplasms | ADAMTS8;KLF2;AP3S2;NUP210;LECT2;PGC;HPX;FABP4 | 8 | 1.47 | 12185 | 0.5003 | 0.691168 |
| MESH:D009203 | Myocardial Infarction | PGC;NUP210;LECT2;HPX;FABP4;ADAMTS8;KLF2;AP3S2 | 8 | 1.47 | 12232 | 0.5003 | 0.691168 |
| MESH:D006330 | Heart Defects, Congenital | KLF2;ADAMTS8;AP3S2;FABP4;HPX;NUP210;PGC;LECT2 | 8 | 1.48 | 12116 | 0.5003 | 0.691168 |
| MESH:D009135 | Muscular Diseases | FABP4;ADAMTS8;KLF2;AP3S2;NUP210;LECT2;PGC;HPX | 8 | 1.48 | 12079 | 0.5003 | 0.691168 |
| MESH:D002471 | Cell Transformation, Neoplastic | PGC;LECT2;NUP210;HPX;FABP4;ADAMTS8;AP3S2;KLF2 | 8 | 1.49 | 12004 | 0.5003 | 0.691168 |
| MESH:D014786 | Vision Disorders | FABP4;AP3S2;ADAMTS8;KLF2;NUP210;HPX;LECT2;PGC | 8 | 1.49 | 12066 | 0.5003 | 0.691168 |
| MESH:D001523 | Mental Disorders | FABP4;HPX;NUP210;PGC;LECT2;ADAMTS8;KLF2;AP3S2 | 8 | 1.5 | 11935 | 0.5003 | 0.691168 |
| MESH:D006333 | Heart Failure | KLF2;ADAMTS8;AP3S2;FABP4;HPX;NUP210;PGC;LECT2 | 8 | 1.51 | 11905 | 0.5003 | 0.691168 |
| MESH:D013921 | Thrombocytopenia | HPX;NUP210;LECT2;PGC;AP3S2;KLF2;FABP4;ADAMTS8 | 8 | 1.51 | 11870 | 0.5003 | 0.691168 |
| MESH:D007565 | Jaundice | AP3S2;KLF2;ADAMTS8;FABP4;HPX;PGC;LECT2;NUP210 | 8 | 1.51 | 11861 | 0.5003 | 0.691168 |
| MESH:D011471 | Prostatic Neoplasms | ADAMTS8;FABP4;KLF2;AP3S2;HPX;NUP210;LECT2;PGC | 8 | 1.51 | 11873 | 0.5003 | 0.691168 |
| MESH:D003875 | Drug Eruptions | AP3S2;ADAMTS8;KLF2;FABP4;HPX;NUP210;LECT2;PGC | 8 | 1.52 | 11793 | 0.5003 | 0.691168 |
| MESH:D006331 | Heart Diseases | LECT2;PGC;NUP210;HPX;FABP4;AP3S2;ADAMTS8;KLF2 | 8 | 1.52 | 11776 | 0.5003 | 0.691168 |
| MESH:D008106 | Liver Cirrhosis, Experimental | AP3S2;ADAMTS8;KLF2;NUP210;LECT2;PGC;HPX;FABP4 | 8 | 1.53 | 11682 | 0.5003 | 0.691168 |
| MESH:D002318 | Cardiovascular Diseases | AP3S2;ADAMTS8;KLF2;NUP210;PGC;LECT2;FABP4;HPX | 8 | 1.56 | 11521 | 0.5003 | 0.691168 |
| MESH:D004244 | Dizziness | NUP210;PGC;LECT2;FABP4;HPX;KLF2;ADAMTS8;AP3S2 | 8 | 1.66 | 10813 | 0.5004 | 0.691168 |
| MESH:D015430 | Weight Gain | KLF2;ADAMTS8;FABP4;AP3S2;PGC;LECT2;HPX;NUP210 | 8 | 1.7 | 10561 | 0.5004 | 0.691168 |
| MESH:D020246 | Venous Thrombosis | HPX;NUP210;PGC;LECT2;AP3S2;FABP4;ADAMTS8;KLF2 | 8 | 1.71 | 10506 | 0.5004 | 0.691168 |
| MESH:D050197 | Atherosclerosis | AP3S2;ADAMTS8;KLF2;HPX;FABP4;PGC;LECT2;NUP210 | 8 | 1.77 | 10118 | 0.5004 | 0.691168 |
| MESH:D009374 | Neoplasms, Experimental | KLF2;ADAMTS8;FABP4;AP3S2;LECT2;NUP210;PGC;HPX | 8 | 1.77 | 10153 | 0.5004 | 0.691168 |
| MESH:D007319 | Sleep Initiation and Maintenance Disorders | KLF2;FABP4;ADAMTS8;AP3S2;HPX;NUP210;PGC;LECT2 | 8 | 1.83 | 9784 | 0.5004 | 0.691168 |
| MESH:D013610 | Tachycardia | NUP210;HPX;PGC;LECT2;FABP4;ADAMTS8;KLF2;AP3S2 | 8 | 2.05 | 8753 | 0.5005 | 0.691168 |
| MESH:D015428 | Myocardial Reperfusion Injury | NUP210;LECT2;PGC;HPX;FABP4;ADAMTS8;KLF2;AP3S2 | 8 | 2.25 | 7963 | 0.5005 | 0.691168 |
